# Extracellular NAD^+^ enhances PARP-dependent DNA repair capacity independently of CD73 activity

**DOI:** 10.1038/s41598-020-57506-9

**Published:** 2020-01-20

**Authors:** Anna Wilk, Faisal Hayat, Richard Cunningham, Jianfeng Li, Silvia Garavaglia, Leila Zamani, Davide M. Ferraris, Peter Sykora, Joel Andrews, Jennifer Clark, Amanda Davis, Laurent Chaloin, Menico Rizzi, Marie Migaud, Robert W. Sobol

**Affiliations:** 1 Mitchell Cancer Institute, University of South Alabama, Mobile, AL 36604 USA; 2 Department of Pharmacology, College of Medicine, University of South Alabama, Mobile, AL 36604 USA; 3 Department of Pharmaceutical Sciences, University of Piemonte Orientale, Largo Donegani 2, 28100 Novara, Italy; 4 Institut de Recherche en Infectiologie de Montpellier (IRIM), Université de Montpellier, CNRS, 34293 Montpellier, France; 5 Present Address: Amelia Technologies, 14676 Rothgeb Drive, Rockville, MD 20850 USA

**Keywords:** Cancer, Cell biology

## Abstract

Changes in nicotinamide adenine dinucleotide (NAD^+^) levels that compromise mitochondrial function trigger release of DNA damaging reactive oxygen species. NAD^+^ levels also affect DNA repair capacity as NAD^+^ is a substrate for PARP-enzymes (mono/poly-ADP-ribosylation) and sirtuins (deacetylation). The ecto-5′-nucleotidase CD73, an ectoenzyme highly expressed in cancer, is suggested to regulate intracellular NAD^+^ levels by processing NAD^+^ and its bio-precursor, nicotinamide mononucleotide (NMN), from tumor microenvironments, thereby enhancing tumor DNA repair capacity and chemotherapy resistance. We therefore investigated whether expression of CD73 impacts intracellular NAD^+^ content and NAD^+^-dependent DNA repair capacity. Reduced intracellular NAD^+^ levels suppressed recruitment of the DNA repair protein XRCC1 to sites of genomic DNA damage and impacted the amount of accumulated DNA damage. Further, decreased NAD^+^ reduced the capacity to repair DNA damage induced by DNA alkylating agents. Overall, reversal of these outcomes through NAD^+^ or NMN supplementation was independent of CD73. In opposition to its proposed role in extracellular NAD^+^ bioprocessing, we found that recombinant human CD73 only poorly processes NMN but not NAD^+^. A positive correlation between CD73 expression and intracellular NAD^+^ content could not be made as CD73 knockout human cells were efficient in generating intracellular NAD^+^ when supplemented with NAD^+^ or NMN.

## Introduction

The tumor microenvironment has the potential to provide critical metabolites to promote tumor cell growth and immune modulation^1^ as well as support cellular metabolism via metabolic coupling^2^ or metabolic plasticity^3,4^. A key energy metabolite in the tumor microenvironment is the hydride exchanger nicotinamide adenine dinucleotide (NAD^+^/NADH) which plays an important role in redox reactions for a number of energy-related and regulatory processes^5–8^. Mammalian cells, in order to sustain intracellular NAD^+^ homeostasis, can preferentially utilize a *de novo* synthesis pathway from L-tryptophan (Trp) or the Preiss-Handler pathway from nicotinic acid (NA), or employ the more effective salvage pathway^9^, which initiates from nicotinamide (NAM), or the nicotinamide riboside (NR) kinase pathway. It is suggested that a source of NAD^+^ and related NAD^+^ metabolites arises from cell lysis at sites of inflammation or tumor cell necrosis^10^, providing substrates for NAD^+^-consuming glycohydrolase ectoenzymes such as CD38 in concert with connexin 43^11^ or NAD^+^-consuming pyrophosphatases such as NPP5^12^.

NAD^+^ is also an essential substrate for signaling and protein modification factors that impact cell death, stress responses and genome stability via mono- or poly-ADP-ribosylation (PARP family proteins)^13^, chromatin status via deacetylation (sirtuins)^14^ and overall functional capacity of mitochondria^15^. Importantly, nuclear/mitochondrial crosstalk is mediated in part by NAD^+^ and NAD^+^ precursors to facilitate genome stability and the cellular response to genotoxic and cytostatic insults^16,17^.

The last few years have opened a new chapter in NAD^+^ biology since a decrease in the cellular NAD^+^ level has been associated with aging and a variety of pathological syndromes including obesity, neurodegenerative diseases, hearing loss as well as cancer^6,18–21^. Additionally, chemotherapeutic agent treatment can decrease NAD^+^ levels and may directly impact the tryptophan pathway^17,22,23^. Furthermore, the plasma NAD^+^ metabolome was shown to be affected by normal aging^24^. These pathological conditions are associated with genome instability, and can be impacted by changes in cellular NAD^+^. As NAD^+^ is a substrate for the DNA repair and DNA damage response signaling enzymes PARP1, PARP2 and PARP3^25^, fluctuations in the cellular levels of NAD^+^ can therefore influence DNA repair mechanisms^26^, modulate chromatin structure^27,28^, regulate transcription^29^, affect telomere function^30^ and impact cell death pathways^15^.

NAD^+^ supplements have been demonstrated to positively impact DNA repair in the context of aging and neurodegeneration in diseases such as Xeroderma pigmentosum complementation group A (XPA)^31^, Cockayne syndrome group B (CSB)^32^, Ataxia-Telangiectasia (A-T) syndrome^33^ as well as in Alzheimer’s disease and other age-related disorders^34^. Defects in DNA repair pathways in these syndromes initiate hyperactivation of PARP1, leading to severe NAD^+^ depletion. Supplementation with NAD^+^ precursors decreased the accumulation of endogenous DNA damage and improved DNA repair capacity^33,35^.

NAD^+^ also has important implications in cancer and its availability affects cell proliferation, invasion and tumor growth^14^. Simultaneously, nicotinamide phosphoribosyl transferase (NAMPT), the rate limiting enzyme in NAD^+^ biosynthesis, is overexpressed in a number of cancers^36–38^ and its expression has been associated with tumor progression in patients^39^, rendering NAMPT an attractive therapeutic target^40^. NAMPT inhibitors such as FK866 and CHS828 demonstrated reasonable efficacy against solid and hematologic cancers in preclinical testing. However, the same inhibitors failed when tested in clinical trials^41–45^. This may indicate that when deprived of NAM as the main NAD^+^ source, cancer cells have an ability to utilize other NAD^+^ biosynthesis pathways^46,47^. NAD^+^ precursors such as Trp, NA and NAM are found in most food, while other precursors such as NR and NMN are detected in plasma, body fluids and milk^48–51^. In a tumor mass, there is an increased risk of hypoxia-induced necrosis and necrotic cells can subsequently become a localized source of NAD^+^ precursors^52^.

In this study, we investigated the role of the extracellular CD73 enzyme in the process of NAD^+^ uptake and biosynthesis from exogenous precursors and in particular, if CD73 status in cancer cells affects DNA repair processes by modulating intracellular NAD^+^ levels. CD73 is an ecto-5′-nucleotidase expressed in a majority of cells and is characterized by dual enzymatic activity. First, it is suggested that CD73 cleaves NAD^+^ to NMN plus adenosine monophosphate (AMP). Second, it has been proposed that the ectonucleotidase activity of CD73 allows for the hydrolysis of both AMP and NMN, leading to the accumulation of adenosine and NR, respectively^47,53,54^. This enzymatic process has been shown using the CD73 bacterial orthologue, *HiNadN*, from *Haemophilus influenza*^54^.

CD73 has been linked with cancer mostly because of its capacity for generating adenosine, as adenosine possesses immunosuppressive potential which affects anti-tumoral T-cell responses^55,56^. Furthermore, multiple reports have correlated CD73′s role in cancer cell adhesion, migration, angiogenesis and invasion to its ability to regulate adenosine levels^57,58^.

In contrast, we focused on the potential involvement of CD73 in modulating NAD^+^ levels in cancer cells to resolve an ongoing debate in the field with regard to a role for CD73 in metabolizing extracellular NAD^+^ and NMN to NR that then could contribute to intracellular levels of NAD^+^^59^. Cancer cells with increased CD73 expression would have an advantage by accessing a broader range of extracellular NAD^+^ precursors for recycling and NAD^+^ biosynthesis, particularly if the tumor microenvironment provided localized NAD^+^ precursors such as NMN that could be used to elevate intracellular NAD^+^ levels^60^. The enzymatic activity of CD73 has been demonstrated in the context of a bacterial study^54^ but importantly there is still a lack of evidence defining an enzymatic activity of CD73 in mammalian cells with regard to NAD^+^ biosynthesis^47,53^. In this study, we used different NAD^+^ precursors, all of which can be utilized for NAD^+^ biosynthesis in cancer cells through individual biosynthetic pathways. We also included a NAMPT inhibitor in order to deplete cells of NAD^+^ and increase the requirement for the uptake of NAD^+^ precursors. Finally, we evaluated the impact of NAD^+^ modulation on DNA damage and DNA repair processes as well as on DNA repair protein complex formation in human cells in the presence or absence of CD73. This study should expand our understanding on the role of CD73 in cancer; but more importantly, its role in NAD^+^ biosynthesis to promote cancer cell growth and chemotherapy resistance by enhancing DNA repair.

## Results

### Intracellular NAD^+^ modulates DNA repair capacity

NAD^+^ is a substrate for DNA repair proteins such as PARP1, PARP2 and PARP3 as well as enzymes that can influence DNA repair capacity such as SIRT1 and SIRT6^15,25,61^. As a substrate for these enzymes, NAD^+^ availability may directly affect DNA repair pathway function as well as modulate chromatin structure to further influence DNA repair capacity. Therefore, we asked if changes in intracellular NAD^+^ content affects the levels of genotoxin-induced DNA damage as well as DNA repair capacity.

**Figure 1:**
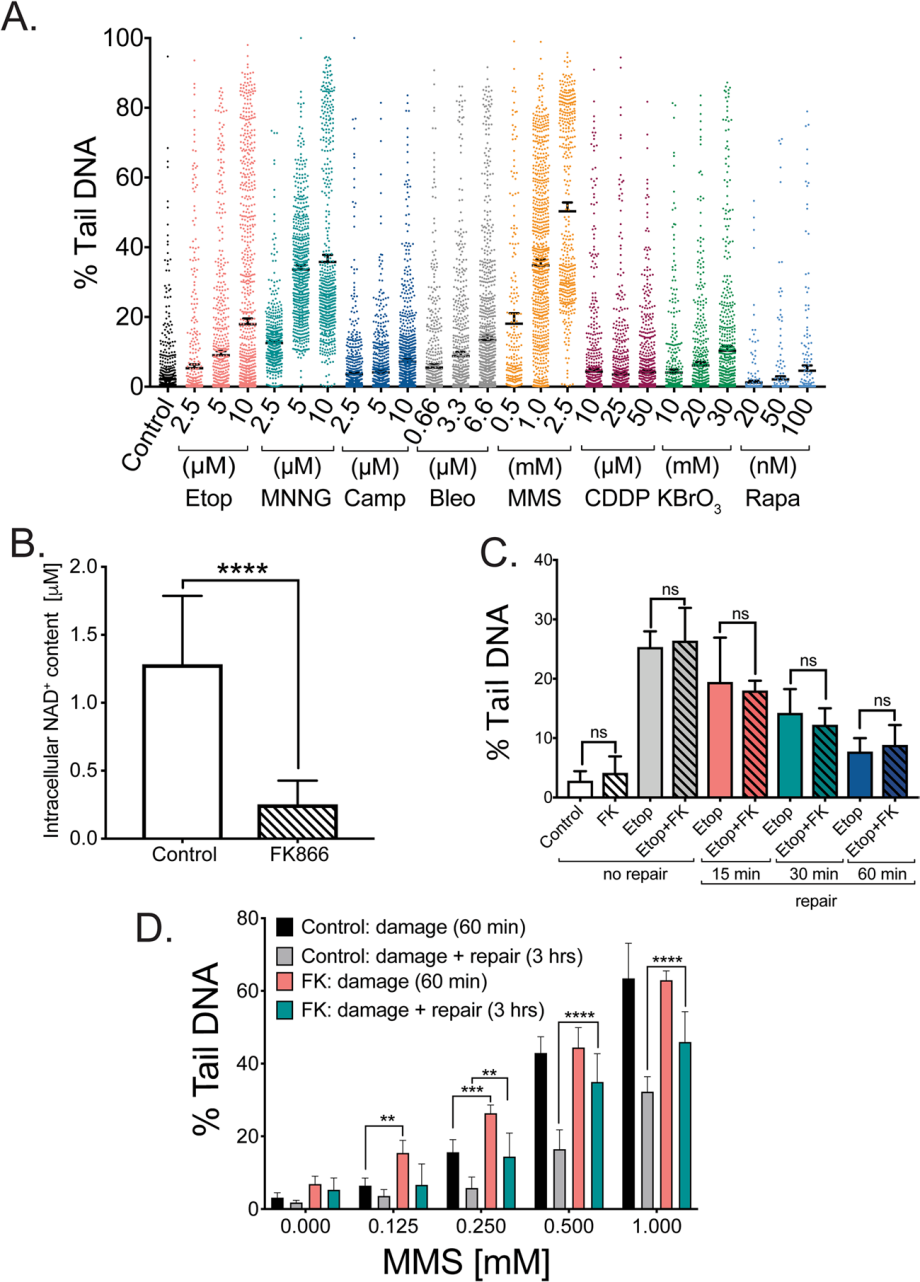
Effect of NAD^+^ depletion on DNA damage and repair in MCF-7 cells as determined using the CometChip assay^62^. **(A)** Level of DNA damage in MCF-7 cells exposed (1 hr) to different concentrations of various DNA damaging agents. **(B)** Level of intracellular NAD^+^ in MCF-7 cells 24 hrs after treatment with FK866 (30 nM) as compared to control. Statistical analysis was conducted via an unpaired t-test (****p < 0.001). **(C)** Level of DNA damage and repair capacity in MCF-7 cells exposed for one hr to etoposide (10 μM) in the presence or absence of FK866 (FK; treatment with FK866 results in a 70–90% decrease in total cellular NAD^+^ levels^16^). **(D)** Level of DNA damage and DNA repair capacity in MCF-7 cells exposed for one hr to different MMS concentrations (0, 0.125, 0.25, 0.5 and 1 mM) in the presence or absence of FK866 (FK; treatment with FK866 results in a 70–90% decrease in total cellular NAD^+^ levels^16^). Data is expressed as % Tail DNA, with standard deviation. Plots are the average of 8–12 wells from two-three independent CometChips; >1000 comets per bar. Statistical analysis was performed using GraphPad Prism 7 and two-way ANOVA followed by *post-hoc* with Tukey’s multiple comparison test (**p < 0.0029, ***<0.0008, ****<0.0001).

We therefore used the next-generation single cell gel electrophoresis assay, the CometChip^62^, to evaluate cellular genomic DNA damage and repair in human cells. As we have reported, this newly developed CometChip system resolves many of the limitations of the traditional comet assay such as reproducibility and variability^62^. The measurement of DNA damage by the CometChip is based on the fact that supercoiled (undamaged) DNA will not migrate in an agarose matrix under an electric field in contrast to fragmented (damaged) DNA which migrates to the positive electrode. The extent and the amount of migrated DNA (% Tail DNA) correlate with the amount of accumulated breaks in the DNA^63^. In these experiments, we used the alkaline CometChip assay, which results in an increase in % Tail DNA for many different types of DNA damage such as alkaline-sensitive base lesions, abasic sites, single-strand and double-strand DNA breaks^64^. As shown in Fig. 1A, the methylating agent methylnitronitrosoguanidine (MNNG), an S_N_1 type alkylating agent, the methylating agent methyl methanesulfonate (MMS), an S_N_2 type alkylating agent, and etoposide, a topoisomerase II inhibitor, induced the highest amount of damage while bleomycin and potassium bromate (KBrO_3_) treatment resulted in a more modest level of DNA damage. The other three compounds (camptothecin, cisplatin (CDDP) and rapamycin) induced a minimal increase in measurable DNA damage over background (approximately 5% Tail DNA) at the indicated doses and time of exposure (1 hr). For the follow-up repair capacity experiments, we selected two agents that generate different classes of DNA damage: etoposide and MMS.

To assess the effect of alterations in the cellular level of NAD^+^ on DNA damage accumulation and DNA repair capacity, we used the NAMPT inhibitor FK866 to deplete the intracellular NAD^+^ pool^16^. The FK866-treatment protocol (24 hrs; 30 nM or 60 nM) results in an 80–90% reduction in the intracellular level of NAD^+^, as we reported^16^ (Fig. 1B). Following FK866 treatment, cells were exposed to damaging agents (1 hr) followed by a repair course whereby the cells were washed to remove the DNA damaging agents and the cells were then incubated in fresh media (at 37 °C) to promote repair of the induced DNA lesions. MCF-7 cells exhibit a very low level of endogenous DNA damage (Fig. 1C, D), which was not significantly affected by NAD^+^ depletion (FK866 treatment). Treatment of MCF-7 cells with etoposide (10 µM) induced approximately 30% Tail DNA, consistent with our previous CometChip analyses^62^. A repair course of 15, 30 and 60 minutes was sufficient for greater than 75% repair, as we have shown for TK6 and Jurkat cells^62^. MCF-7 cells presented with time-dependent repair of the etoposide-induced DNA damage in both the FK866-treated and untreated cells, indicating that NAD^+^ status did not affect the amount of induced damage nor the DNA repair capacity in response to etoposide treatment (Figs. 1C and S1A). Correspondingly, we find that PARP1-KO cells show no difference, as compared to control cells, in the rate of repair of DNA damage induced by etoposide (J. Li and R.W.Sobol, *Manuscript in Prep*).

MMS treated MCF-7 cells showed a dose dependent accumulation of DNA damage but in contrast to the etoposide-induced damage, MMS-induced DNA damage required a longer time (3 hrs) for repair of the induced DNA lesions (Figs. 1D and S1B). At low MMS concentrations (0.125 and 0.25 mM), MCF-7 cells demonstrated a greater level of DNA damage in the absence of NAD^+^ than in the presence of NAD^+^; however, the level of MMS-induced DNA damage was the same at higher MMS concentrations regardless of the NAD^+^ content (Fig. 1D). As might be expected by the requirement for PARP1 in BER-mediated repair of MMS-induced DNA damage^65^, NAD^+^ depletion significantly suppressed the repair of the DNA damage, with as much as a 40% lower rate of repair when NAD^+^ was depleted (Figs. 1D and S1B).

### Cancer cells express elevated mRNA levels for genes involved in the NAD^+^ salvage pathway

We next evaluated the genes required for cells to maintain sufficient NAD^+^ levels that would be critical to support metabolism, as we have shown^16^, as well as maintain genome stability (Fig. 1). We performed a comparative analysis of mRNA expression of the genes involved in NAD^+^ metabolism in MCF-7 cells, plus an additional eight cancer cell lines, including LN428, T98G, A375, G09607, HCT116, MDA-MB-231, NCI.H460 and U87MG (Table 1). We divided the genes into three categories; (i) enzymes involved in *de novo* NAD^+^ biosynthesis, (ii) enzymes involved in the salvage pathway and (iii) NAD^+^ consuming enzymes (Table 1). All the cell lines strongly expressed most genes involved in the salvage pathway as compared to those involved in the *de novo* pathway, most prominently seen for NAMPT and purine nucleoside phosphorylase (PNP). In addition, kynureninase (KYNU) and tryptophan-2,3-dioxygenase (TDO2) of the *de novo* pathway were also elevated. The elevated expression of NAMPT and PNP is consistent with multiple reports and clinical studies showing elevated levels of these enzymes in several cancers^36,37,44,66^. NNMT, the nicotinamide N-methyltransferase reported to be elevated in many cancers^67^ and cancer-associated fibroblasts^68^, is also shown elevated in most of the cell lines evaluated (Table 1). Further, all three categories of enzymes are shown to be amplified in breast cancer tumors^69,70^, as shown in Supplementary Fig. S2A–C.

**Table 1:** –mRNA expression of genes involved in NAD^+^ metabolism.

	Gene Symbol	Protein symbol	LN428	T98G	A375	G09607	HCT116	MCF-7	MDA-MB-231	NCI.H460	U87MG
De novo pathway	AFMID	AFMID	40.89	45.79	33.45	66.57	204.48	55.25	51.44	73.62	57.21
	HAAO	HAAO	29.49	37.08	25.96	24.75	25.32	25.01	26.06	27.74	26.41
	IDO1	IDO1	14.88	18.55	10.28	10.23	11.46	11.02	11.57	10.55	10.92
	IDO2	IDO2	29.80	33.33	28.09	26.69	31.94	26.52	28.48	31.50	28.45
	KMO	KMO	83.45	14.30	7.36	7.41	7.82	7.07	7.65	8.99	7.57
	KYNU	KYNU	57.84	2249.41	170.52	7.52	7.02	1983.97	483.52	2515.09	698.91
	QPRT	QPRT	52.12	38.65	29.42	19.19	17.89	178.43	18.17	19.16	64.10
	TDO2	TDO2	17.21	33.97	11.03	11.17	8.72	8.01	13.09	297.70	607.43
Salvage pathway	NMRK1	NRK1	17.32	15.70	13.43	107.94	19.98	51.06	15.13	92.69	64.43
	NMRK2	NRK2	17.81	17.06	14.38	13.60	14.25	13.87	15.07	13.26	13.58
	NADK	NADK	277.26	122.72	282.80	245.63	275.73	324.26	168.87	174.61	206.54
	NADSYN1	NADSYN1	55.78	89.77	52.15	83.67	56.34	227.16	88.74	87.07	147.14
	NAMPT	NAMPT	576.38	806.05	835.90	306.31	320.68	205.40	690.60	2235.86	1237.03
	NAPRT	NAPRT1	30.58	43.24	34.38	160.61	254.06	123.02	118.04	42.58	33.53
	NMNAT1	NMNAT1	40.97	25.39	57.95	49.21	52.60	46.96	39.11	27.37	73.35
	NMNAT2	NMNAT2	61.85	66.41	36.01	63.98	19.56	38.67	132.46	78.27	197.11
	NMNAT3	NMNAT3	47.09	31.96	18.85	18.99	32.23	24.09	20.71	20.94	19.06
	PNP	PNP	851.34	221.99	394.01	656.57	648.50	634.86	1424.01	1122.68	383.69
NAD^+^ consuming enzymes	BST1	CD157	19.21	8.86	8.31	138.09	9.43	7.45	11.74	111.67	25.21
	CD38	CD38	10.62	9.63	9.04	622.73	8.14	8.72	8.74	8.36	8.41
	ENTPD1	CD39	17.57	18.20	17.15	16.72	19.28	16.32	21.65	18.77	16.39
	NNMT	NNMT	448.21	731.95	1127.63	565.88	24.03	18.71	26.03	154.81	410.01
	NT5E	CD73	1504.20	19.21	689.83	1438.59	469.60	90.63	2247.53	379.95	1033.97
	PARP1	PARP1	518.96	604.91	398.42	1033.13	639.85	415.14	680.80	794.18	129.65
	PARP2	PARP2	267.44	206.39	206.23	299.33	412.53	266.18	385.40	353.05	119.51
	PARP3	PARP3	196.23	177.90	163.03	238.20	127.02	38.35	141.02	116.70	239.57
	SIRT1	SIRT1	395.87	311.12	182.53	360.42	411.73	326.82	342.83	341.26	313.82
	SIRT2	SIRT2	332.45	228.72	230.47	255.80	250.11	513.48	338.45	323.30	299.61
	SIRT3	SIRT3	208.19	278.25	94.12	98.92	169.51	87.97	120.82	149.62	129.08
	SIRT4	SIRT4	18.43	21.91	14.83	14.85	17.95	13.93	14.91	14.86	13.02
	SIRT5	SIRT5	62.25	47.84	16.97	36.65	58.62	47.85	52.25	31.19	30.79
	SIRT6	SIRT6	63.69	67.00	64.35	57.34	65.25	75.76	60.34	60.45	70.75
	SIRT7	SIRT7	268.57	249.30	116.31	225.22	271.26	263.94	188.41	180.28	236.76
	TNKS	TNKS	71.42	196.13	155.65	92.31	165.27	87.94	77.52	78.46	220.74
	TNKS2	TNKS2	28.17	128.73	96.43	137.27	175.87	84.43	109.39	103.54	121.72

Regarding the group of NAD^+^ consuming enzymes, the majority of genes that displayed high mRNA expression were PARP-family and sirtuin-family member proteins. However, the gene with the most varied expression was the ectoenzyme CD73 (NT5E)^71^. Given that the role of PARPs and sirtuins is well documented in a variety of cancers^14^, we decided to focus on the CD73 ectoenzyme. CD73, together with CD38 (gene = CD38, an NADase/glycohydrolase/ADP-ribosyl cyclase) and CD157 (gene = BST1; an NADase and ADP-ribosyl cyclase), are surface enzymes reported to consume NAD^+^ as a substrate for their enzymatic activity^10,72,73^. While the majority of cell lines expressed low levels of CD38 (8 out of 9) and CD157 (7 out of 10), most of the cells analyzed in this study expressed high levels of CD73 mRNA, with the exception of T98G cells. Of the cell lines under study herein, CD38 expression was not detected at the mRNA or protein level (Supplementary Fig. S2D, E). In the present study, we focused primarily on the role of CD73 in NAD^+^ metabolism, which could impact intracellular NAD^+^ content and therefore genomic stability of cancer cells. To this end, four cell lines were selected for our investigation: two glioma cell lines (LN428 and T98G) and two breast cancer cell lines (MDA-MB-231 and MCF-7). These cell lines express varying levels of CD73, representing, as such, the variances observed in the cell lines examined (Table 1).

### Analysis of NMN, NAD^+^ and NADH catabolic activity by recombinant, human CD73

**Figure 2:**
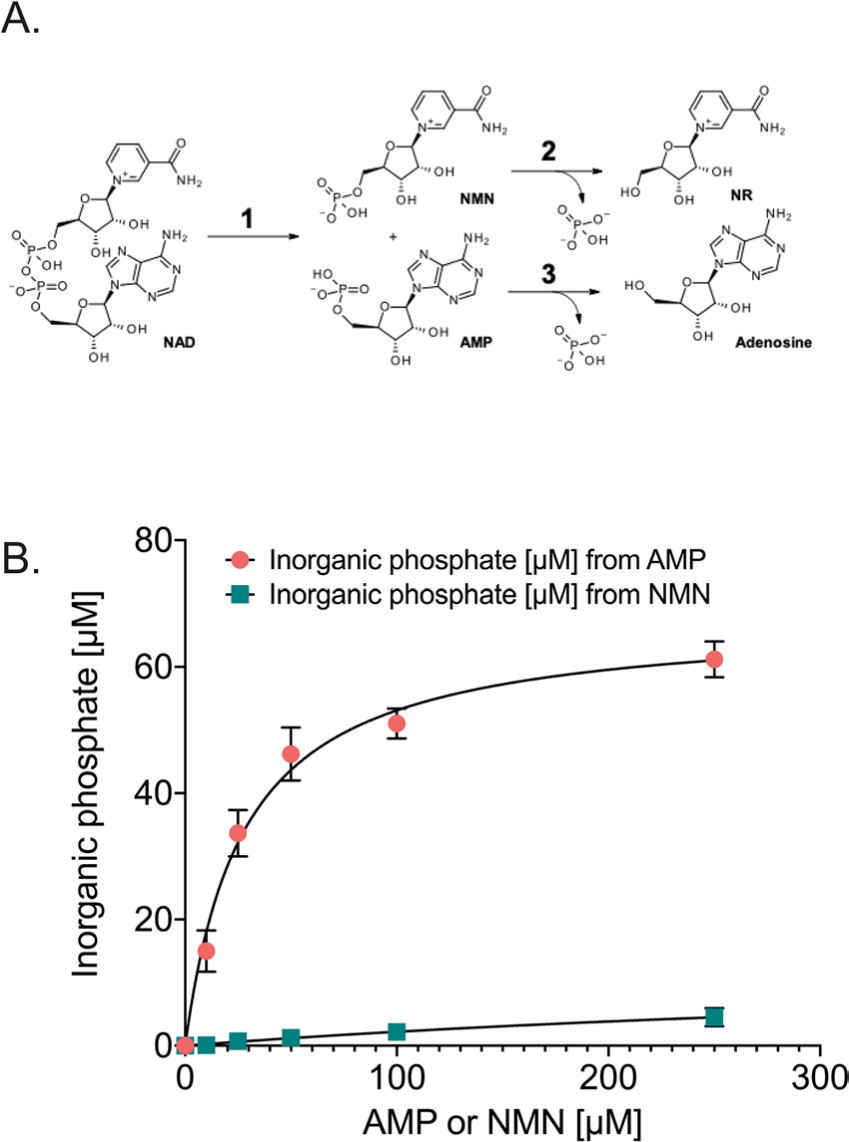
Human, recombinant CD73 activity analysis for the hydrolysis of NMN, NAD^+^ and NADH. **(A)** Scheme showing the structure of NAD^+^ and conversion to AMP (bottom) and then to adenosine with loss of inorganic phosphate and to NMN (top) and then to NR. **(B**) Recombinant CD73 (rCD73; soluble form, residues 27–549 including a His-tag at the C-terminus) was purified and evaluated for enzymatic activity measured in the presence of the natural substrate, AMP, or in the presence of NMN. There was no detectable activity against NAD^+^ or NADH (not shown).

The *H. influenzae* enzyme *HiNadN*, considered to be the ortholog to the human CD73 enzyme, was previously shown to hydrolyze NAD^+^ and NMN when evaluated by HPLC and colorimetric assays^54,74–76^, suggesting that the human enzyme (CD73) may also catabolize these extracellular metabolites, processing NAD^+^ to AMP and NMN and then hydrolyzing both AMP and NMN to adenosine and nicotinamide riboside (NR), respectively (Fig. 2A). Commercially available preparations of human CD73 were all shown to be inactive (Supplementary Fig. S3A–D). NADH, just like NAD^+^, is likely to be present in the microenvironment of a tumor and potentially be hydrolyzed to the reduced form of NR, NRH, a known precursor to NAD^+^^77^. Therefore, to directly evaluate the capacity of the human CD73 enzyme to catabolize NMN, NAD^+^ or NADH, the soluble form of recombinant CD73 (residues 27–549 including a His-tag at the C-terminus) was produced in Sf9 insect cells and purified as previously described^78^. The purified rCD73 is highly active against AMP yet inactive against NAD^+^ or NADH and very poorly active against NMN (Fig. 2B). Similarly, we show that the *H. influenzae* enzyme *HiNadN* is able to effectively catabolize NAD^+^ yet does not process NMN when measured by ^31^P NMR (Supplementary Fig. S4). Our *in vitro* biochemical analysis of the recombinant purified enzymes supports a role for the bacterial enzyme *HiNadN* in the processing of NAD^+^ with no evidence for NAD^+^ hydrolysis catalyzed by the recombinant, purified human CD73 enzyme. Further, we find that the recombinant, purified human CD73 enzyme processes NMN extremely poorly (Fig. 2B).

### Impact of differential CD73 expression on the intracellular NAD^+^ content in human cancer cell lines

**Figure 3:**
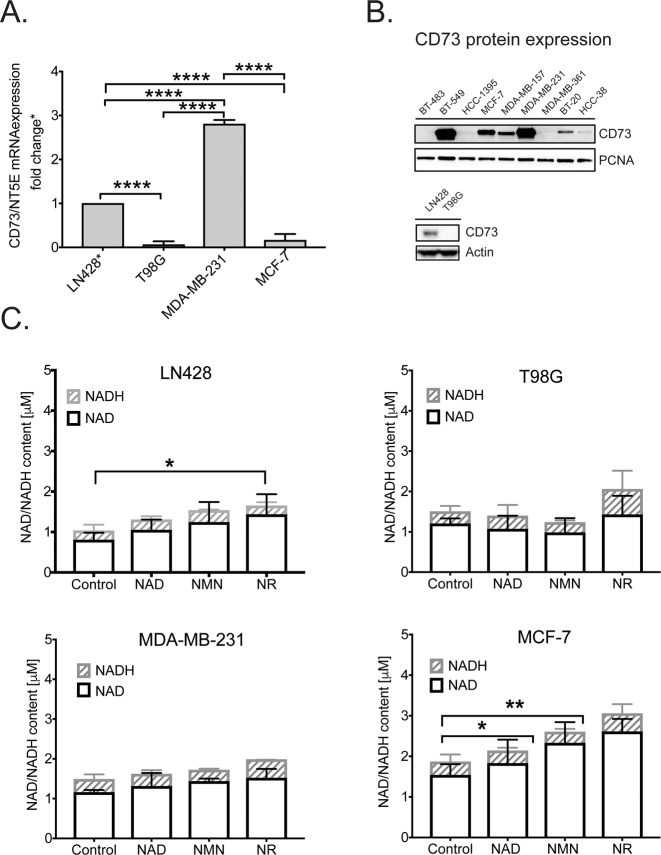
Comparative analysis of CD73 expression in human cancer cell lines and its effect on NAD^+^ biosynthesis. **(A)** Measurement of mRNA expression for the CD73/NT5E gene in cancer cell lines, as determined by qRT-PCR analysis, normalized to the expression of human β-actin mRNA via the ∆∆CT method. **(B)** Immunoblot analysis of the expression of CD73 in nine breast cancer cell lines, including the MCF-7 and MDA-MB-231 cells used herein (top panel) and two glioma cell lines, including the LN428 and T98G cells used herein (bottom panel). PCNA was used as a loading control for the top panel and actin was used as a loading control for the bottom panel. See Supplement Fig. S2F for the expression of NRK1 in the breast cancer cell lines analyzed from the same cell lysates. **(C)** Total intracellular NAD levels (NAD^+^ and NADH) in each of the four cancer cell lines cultured in the presence of NAD^+^, NMN or NR (100 μM) for 24 hrs. Statistical analysis was performed using GraphPad Prism 7 and two-way ANOVA followed by *post-hoc* test with Tukey’s correction (ns = not significant, *p = 0.0419, **p = 0.0032).

Given that NAD^+^ is not hydrolyzed by recombinant human CD73 and is very poorly active against NMN (Fig. 2B), this raised the question as to whether CD73 plays any role at all in metabolizing extracellular NAD^+^ and therefore in regulating the intracellular level of NAD^+^ in human cells. We confirmed the expression level of CD73 mRNA and protein in four cancer cell lines by qRT-PCR and immunoblot analysis, respectively. The breast cancer cell line MDA-MB-231 displayed extremely high levels of CD73 at both the mRNA and protein level (Fig. 3A, B). The glioblastoma cell line LN428 also displayed high levels of CD73 expression but significantly lower compared to the MDA-MB-231 cells (Fig. 3A, B). The breast cancer cell line MCF-7 expressed CD73 mRNA and protein yet the glioblastoma cell line T98G exhibited very low to almost undetectable levels of CD73 (Fig. 3A, B). Among the human cancer cell lines we have evaluated, the expression of CD73 is highly varied as compared to other proteins involved in NAD^+^ metabolism, such as NRK1 (Figs. 3B and S2F).

Therefore, we first examined whether CD73 is indeed responsible for hydrolyzing NAD^+^, as shown in the scheme in Fig. 2A, and if the variations in expression impact the intracellular level of NAD^+^. To this end, we measured the total NAD pool (NAD^+^ plus NADH) in all four cell lines following addition (100 μM, 24 hrs) of nicotinamide adenine dinucleotide (NAD^+^), nicotinamide mononucleotide (NMN) or nicotinamide riboside (NR) to the culture media. The basal (control) NAD^+^ and NADH content (as per the colorimetric analysis) was similar among all the cell lines (Fig. 3C), although there was slight variation. LN428 cells had the lowest levels of NAD^+^ (0.80 µM) and NADH (0.26 µM) while MCF-7 cells had the highest levels of NAD^+^ (1.51 µM) and NADH (0.35 µM). While the addition of all of the precursors to the culture media increased NAD^+^ levels in all cell lines (Fig. 3C), only NR affected NADH levels statistically. Interestingly, the increase in NADH following NR supplementation in the 4 cell lines (LN428, T98G, MDA-MB-231 and MCF-7) does not seem to correlate with the corresponding mRNA levels of PNP (Supplementary Fig. S2G). Importantly, despite highly variable levels of CD73 among the four cell lines, no correlation between CD73 expression level, NAD^+^ or NMN supplementation and intracellular NAD^+^ content was observed. Interestingly, MCF-7 cells, which express a lower level of CD73 as compared to the MDA-MB-231 cells (Table 1, Fig. 3A,B) displayed the highest basal intracellular NAD^+^ level and in the presence of NAD^+^ and the vitamin B_3_ precursors, NMN or NR. This is consistent with the report that MCF-7 is an “NAD keeper” as measured by flux analysis^79^.

### NAMPT inhibition accelerates uptake of NAD^+^ precursors while CD73 inhibition does not affect NAD^+^ biosynthesis

The cancer cell lines under study did not show a correlation between the level of CD73 expression and the intracellular NAD^+^ content when exposed to NAD^+^ precursors in the absence of stress. We therefore next introduced NAMPT inhibitor treatment to disturb cellular NAD^+^ homeostasis. Treatment with FK866, a potent NAMPT inhibitor^41^, is expected to deplete intracellular NAD^+^, as shown in Fig. 1B, and accelerate an uptake of NAD^+^ precursors: NAM, NR and NMN.

**Figure 4:**
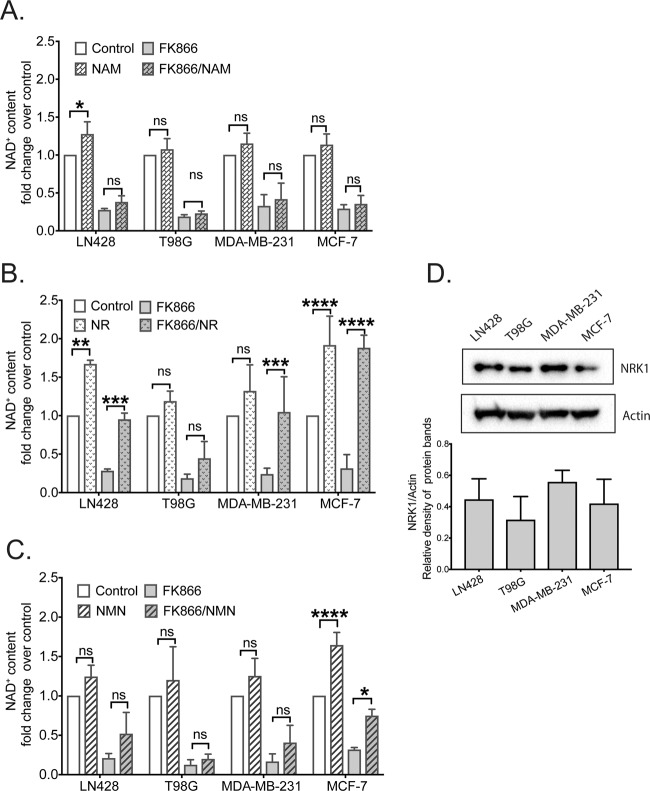
Effect of NAMPT inhibition on intracellular NAD^+^ content in cancer cell lines with different basal levels of CD73 protein. **(A)** Intracellular NAD^+^ levels in cells treated with FK866 (30 nM) and/or nicotinamide (NAM) (100 μM), as compared to untreated controls. **(B)** Intracellular NAD^+^ levels in cells treated with FK866 (30 nM) and/or nicotinamide riboside (NR) (100 μM), as compared to untreated controls. **(C)** Intracellular NAD^+^ levels in cells treated with FK866 (30 nM) and/or nicotinamide mononucleotide (NMN) (100 μM), as compared to untreated controls. **(D)** Top panel: Representative immunoblot analysis of NRK1 expression to correlate the expression of NRK1 in four cancer cell lines (see Supplement Fig. 3E for additional immunoblot figures). Bottom panel: Densitometry analysis of 3 independent immunoblots, performed using Image Lab software. Statistical analysis was performed using GraphPad Prism 7 - one or two-way ANOVA followed by *post-hoc* test with Tukey’s correction was used (ns = not significant, *p = 0.0176, **p = 0.0018, ***p = 0.0009 or ****p < 0.0001).

In the absence of FK866, supplementation of NAM, NR or NMN induced an increase in NAD^+^ levels in only a few of the cell lines. Supplementation of NAM (24 hrs, 100 µM) to the LN428 cell culture media resulted in a modest increase in the basal intracellular level of NAD^+^ with no significant change in the other three cell lines (Fig. 4A). Further, supplementation of NR (24 hrs, 100 µM) caused a significant increase in intracellular NAD^+^ content in LN428 and MCF-7 cell lines with no significant impact on the T98G and MDA-MB-231 cell lines (Fig. 4B), again consistent with a reduced turn-over of the NAD^+^ pool by NAD^+^-consuming enzymes in MCF-7 cells compared to MDA-MB-231 cells^79^. Finally, supplementation of NMN (24 hrs, 100 µM) to the culture media only increased the basal intracellular level of NAD^+^ in the MCF-7 cell line, with no significant alteration in the other three cell lines (Fig. 4C). As expected, when cells were supplemented with NAM in the presence of FK866, NAM was not able to rescue NAD^+^ levels since its metabolism entirely depends on NAMPT activity. In contrast, NR supplementation resulted in the greatest increase in NAD^+^ levels in the presence of FK866. Although there was no significant increase in T98G cells, LN428, MDA-MB-231 and MCF-7 cells all reported a strong increase in NAD^+^ levels although never higher than the cells supplemented with NR in the absence of FK866 (Fig. 4B). However, NR metabolism does not depend on CD73 activity. The addition of NMN was also able to rescue NAD^+^ levels but not as efficiently as NR in FK866 treated cells and reached significance only in the MCF-7 cell line. Notably, even when cells are stressed by NAD^+^ deprivation, the high level of CD73 expression, as seen in the MDA-MB-231 cells, did not affect the uptake of precursors or the increase in NAD^+^ biosynthesis (Fig. 4C). In summary, there is a lack of any significant difference in NAD^+^ content between cells with undetectable levels of CD73 (such as T98G), cells with CD73 expression (MCF-7) or cells with high or very high levels of CD73 (such as LN428 and MDA-MB-231, respectively). We also tested if inhibiting CD73, using the competitive inhibitor adenosine 5′-(α,β-methylene) diphosphate (APCP), would impact intracellular NAD^+^ content. In-line with our cell line analyses (Fig. 4), APCP did not cause major changes in NAD^+^ levels and inhibition of CD73 exerted no effect on cellular NAD^+^ content in combination with FK866 treatment (Supplementary Fig. S5A–D).

We therefore reasoned it was then critical to determine which enzyme in the NAD^+^ biosynthesis pathway could be responsible for such a strong effect on NAD^+^ biosynthesis. Given that NRK1 and NRK2 are the kinases that phosphorylate NR to produce NMN^50^, they represented the likely candidates that may influence NAD^+^ biosynthesis in cells supplemented with NR. Not surprisingly, a qRT-PCR assessment revealed no detectable levels of *NRK2* mRNA expression in MCF-7 cells (data not shown), since *NRK2* is exclusively expressed in muscle tissue^80^. Similarly, NRK1 protein levels in the four tested cell lines, as assessed by immunoblot analysis, did not correlate with the measured NAD^+^ levels (Fig. 4D). Overall, these results did not explain the mechanism by which MCF-7 cells were the most effective at utilizing NAD^+^ precursors.

### Kinetic analysis of cell culture supernatants reveals the products of CD73 enzymatic activity

Direct enzymatic analysis of the recombinant human CD73 enzyme (rCD73) revealed that purified rCD73 is highly active against AMP yet inactive against NAD^+^ or NADH and very poorly active against NMN, if at all (Fig. 2B). Further, we found that NMR analysis of the bacterial orthologue of CD73, *HiNadN*, revealed that *HiNadN* can hydrolyze NAD^+^ to NMN but cannot metabolize NMN to NR. Therefore, to enhance our analysis of human cells expressing CD73, we performed NMR analysis of cell culture supernatants to determine if the supplemented NAD^+^ precursors were subjected to CD73-mediated enzymatic processing. We focused on the MCF-7 cell line since, in the presence of NAD^+^ precursors, only MCF-7 cells were able to overcome CD73 and NAMPT inhibition to restore NAD^+^ levels (Supplementary Fig. S5A–E**)**. Therefore, we treated MCF-7 cells for 24 hrs with NAD^+^ supplements and then compared the content of the cell culture supernatants to media containing precursors that had never been exposed to cells. To our surprise, incubation in media alone induced hydrolysis of the NAD^+^ precursors to the same degree as in the presence of MCF-7 cells. This suggested that the media or one of the media components might impact the stability of the NAD^+^ precursors.

**Figure 5:**
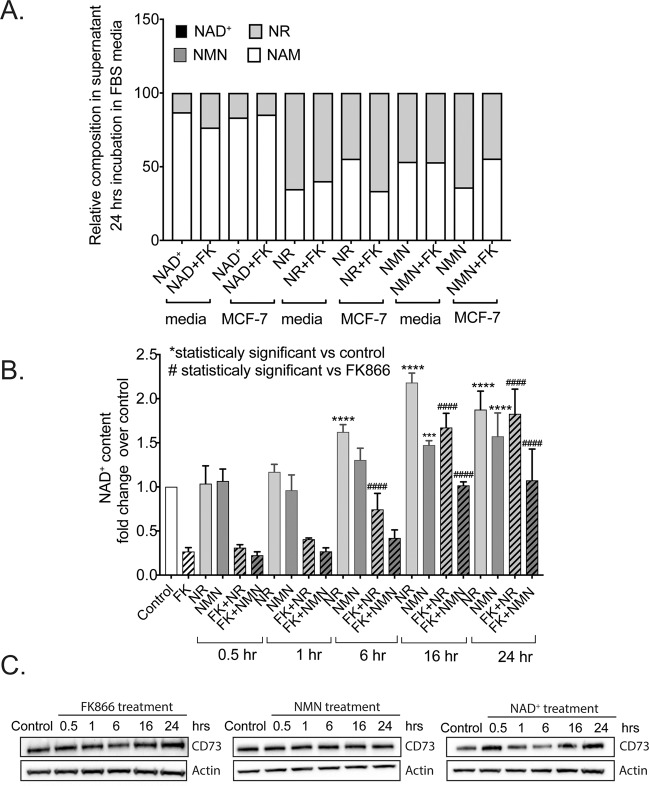
Assessment of the time dependent changes of extracellular NAD^+^ and NAD^+^ metabolite composition and intracellular NAD^+^ levels following metabolite supplementation and/or NAMPT inhibition. **(A)** NMR analysis of the composition of NAD^+^/NAD^+^-metabolites in MCF-7 cell supernatants from cells exposed to NAD^+^ for 24 hrs. **(B)** Measurements of intracellular NAD^+^ content in MCF-7 cells exposed to NAD supplements (NMN and NR) and/or the NAMPT inhibitor, FK866, at different time points after supplement addition. Statistical analysis was performed using GraphPad Prism 7 - one-way ANOVA followed by *post-hoc* test with Tukey’s correction was used. (ns =  not significant, ***p = 0.0004 or ****p < 0.0001) **(C)** Immunoblot analysis to evaluate changes in CD73 expression impacted by the treatment of MCF-7 cells with FK866 (30 nM), NMN (100 μM) or NAD^+^ (100 μM) for times ranging from 0.5 to 24 hrs, as compared to the untreated control, as indicated.

Upon 24 hrs incubation of MCF-7 cells with NAD^+^, NR or NMN in the presence or absence of FK866 (FK), we only detected NR and NAM in the supernatants from all of the tested samples (Fig. 5A). The ratio between NR and NAM was different when compared to the treatments with NAD^+^, NR or NMN but very similar when comparing ‘media only’ with ‘media exposed to cells’. Additionally, the presence of FK866 did not impact the composition or distribution of NR and NAM in the tested samples. At the same time, the presence of NR in the cell supernatants could indicate that hydrolysis of NMN to NR took place. The presence of NAM could imply the enzymatic activity of CD157 or CD38. However, MCF-7 cells do not express these consuming enzymes, as shown in Table 1 and we found no detectable levels of CD38 mRNA or protein (Supplementary Fig. S2D,E).

Next, we measured the intracellular NAD^+^ content in MCF-7 cells at different time points in order to determine how fast the cells would utilize the available precursors for NAD^+^ biosynthesis. We measured NAD^+^ levels in MCF-7 cells exposed to NMN and NR as early as 30 min, followed by 1, 6, 16 and 24 hrs. In addition, we also treated cells with FK866 (FK) for 24 hrs, inducing NAD^+^ deprivation to intensify the uptake of NAD^+^ precursors. At early time points (30 min and 1 hr), the NAD^+^ content in cells treated with the supplements was similar to the untreated MCF-7 cells. In-line with our earlier analysis, 24-hr treatment with FK866 depleted ~75% of the intracellular NAD^+^ and short-term supplementation with NMN or NR did not impact NAD^+^ levels (Fig. 5B).

At 6 hrs post treatment, we observed an increase in NAD^+^ content due to NR supplementation. Finally, after 16 and 24 hrs of treatment, we observed a significant increase in NAD^+^ content upon both NR and NMN supplementation. The presence of FK866 augmented the differences between the conditions, showing a strong effect of NAD^+^ deprivation on the uptake of NAD^+^ precursors (Fig. 5B). Importantly, at 16 and 24 hrs, MCF-7 cells showed an increase in NAD^+^ from NMN uptake. We also tested if NAD^+^ depletion and NAD^+^ supplementation affect the status of CD73 at the protein level. To do so, we exposed MCF-7 cells to NMN and NAD^+^, rather than NR, since their metabolism should depend on CD73 enzymatic activity. We also tested the effect of the FK866 inhibitor on CD73 protein levels in MCF-7 cells. The presence of the NAD^+^ precursors, as well as FK866 treatment, did not affect the level of CD73 protein at any time point (Fig. 5C). However, it is noted that these experiments excluded the potential regulation of CD73 activity by the level of intracellular NAD^+^ or the presence of extracellular NAD^+^ precursors.

### MCF-7/CD73 knockout cells are efficient in hydrolyzing NAD^+^ to NMN and NR

**Figure 6:**
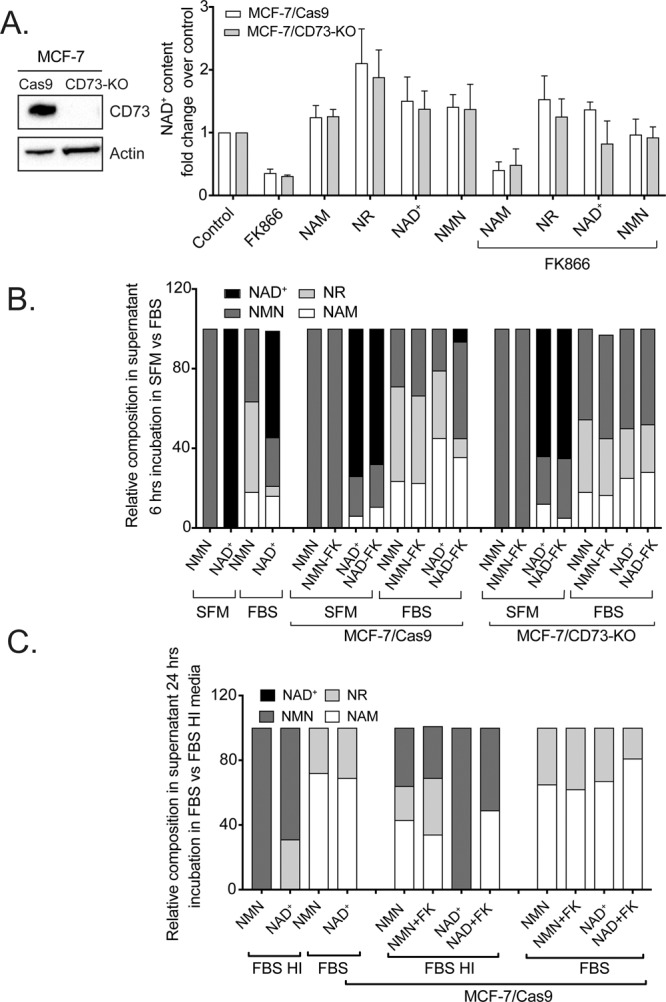
Effect of CD73 knockout on the uptake of NAD^+^ precursors and on NAD^+^ biosynthesis. **(A)** Left panel: Immunoblot analysis of CD73 expression in MCF-7/Cas9 and MCF-7/CD73-KO cells; actin is shown as a loading control. Right panel: Comparative analysis of intracellular NAD^+^ levels in both cell lines exposed to different NAD^+^ precursors (100 μM) in the absence or presence of the NAMPT inhibitor, FK866 (30 nM). **(B)** NMR analysis to define the distribution of NAD^+^ and NAD^+^ metabolites (100 μM) in cell culture media 6 hrs after supplementation of NMN or NAD^+^ to cell-free serum-free media (SFM), cell-free media supplemented with fetal bovine serum (FBS) or when added to SFM or FBS in the presence of MCF-7/Cas9 or MCF-7/CD73-KO cells in the presence or absence of FK866 (30 nM). **(C)** NMR analysis to define the distribution of NAD^+^ and NAD^+^ metabolites in cell culture media 24 hrs after supplementation of NMN or NAD^+^ to MCF-7/Cas9 cells in the presence or absence of FK866. Cells were exposed to media supplemented with fetal bovine serum (FBS) or heat inactivated fetal bovine serum (FBS-HI) and compared to NMN and NAD^+^, which have never been exposed to cells.

To more effectively probe the requirement for CD73 in NAD^+^-precursor uptake, we developed an isogenic system by creating a human MCF-7/CD73-KO cell line using the CRISPR/Cas9 editing system^81,82^. The absence of CD73 protein was confirmed by immunoblot analysis where we compared MCF-7/CD73-KO cells to the isogenic MCF-7/Cas9 cells (Fig. 6A, left panel). This isogenic cell system allows us to exclude the impact of CD73 on NAD^+^ metabolism and helps to address the data showing hydrolysis of NAD^+^, NR and NMN in media only as shown in Fig. 5A.

We then utilized our control (MCF-7/Cas9) and isogenic CD73 deficient (MCF-7/CD73-KO) human cells to determine if the loss of CD73 impacts the uptake of NAD^+^ or NAD^+^-precursors sufficiently to modulate the level of intracellular NAD^+^. As in the previous analyses, we also exposed cells to the NAMPT inhibitor (FK866; 24 hrs, 30 nM) to increase the uptake of the NAD^+^ precursors. MCF-7/Cas9 and MCF-7/CD73-KO cells demonstrated a very similar increase in NAD^+^ content when exposed to all four precursors, with NR supplementation driving the largest increase in NAD^+^ levels (as compared to control), regardless of FK866 treatment (Fig. 6A). For both cell lines, NAM supplementation in the presence of FK866 could not restore NAD^+^ (as expected) since NAM metabolism requires NAMPT enzymatic activity. When cells were supplemented with NR, which should not rely on CD73 for uptake, we observed similar levels of NAD^+^ in both the control (MCF-7/Cas9) and isogenic CD73 deficient (MCF-7/CD73-KO) cells. This may be predictable since there are known nucleoside transporters likely involved in transporting NR or NMN^83,84^. Finally, when we supplemented both cell lines with NAD^+^ or NMN, each suggested as substrates for CD73^54^, there was an equivalent level of intracellular NAD^+^, for both the MCF-7/CD73-KO and MCF-7/Cas9 cell lines. However, the difference was slightly more pronounced when cells were treated with FK866. Once NAMPT was inhibited (with FK866), NMN supplementation completely complemented the NAD^+^ levels in the CD73 deficient cells (MCF-7/CD73-KO) whereas NAD^+^ supplementation resulted in a slight, albeit non-significant, reduction in NAD^+^ complementation (Fig. 6A). Therefore, these studies would suggest that CD73 does not participate in NAD^+^ or NMN uptake and metabolism. Importantly, expression of the proposed NMN transporter (Slc12a8) was found to be very low in all the cell lines under study here, with the expression level in MCF-7 cells effectively below detection, as measured by qRT-PCR (Supplementary Fig. S5F).

Next, we performed NMR analysis of cell supernatants (media) collected from MCF-7/Cas9 and MCF-7/CD73-KO cells to evaluate the catabolism of the supplemented NAD^+^ and NAD^+^-precursors. The cell supernatants were collected after 24 hrs of incubation with vitamin B_3_ precursors as well as the FK866 (FK) inhibitor. Overall, the distribution of precursors between the Cas9 (control) and CD73-KO cell lines showed high similarity. Specifically, supplementation with NR or NR+FK, showed ~50% of the NR being metabolized into NAM, while supplementation with NMN and NMN+FK gave about 40–50% NAM, with NR being the second largest component and a small portion of NMN present in all conditions, with the exception of MCF-7/Cas9 treated with NMN alone. These data suggest robust conversion of NMN to NR and further, NR to NAM, in both CD73 positive and negative cell lines. This confirms that CD73 enzymatic activity is not involved in NMN metabolism in MCF-7 cells since the CD73-KO cells showed the same composition and distribution of the precursors as the MCF-7/Cas9 cells. Finally, supernatants from cells incubated with NAD^+^ or NAD^+^+FK did not show any non-metabolized NAD^+^ (in both cell lines). Instead, we observed NR and NAM as the major components, with a small amount of NMN present. Patterns for both cell lines looked comparable, although MCF-7/Cas9 demonstrated stronger enzymatic activity towards NMN and NAD^+^ when cells where incubated with precursors alone but not when they were co-exposed to the FK866 inhibitor (Supplementary Fig. S5E).

The detection of NAM and NR in the media supplemented with NAD^+^ but never exposed to the cells (Fig. 5A) as well as the contradictory results of complete metabolism of NAD^+^ precursors (Supplementary Fig. S5E) suggested that some of the NAD^+^ precursors were not as stable in media containing fetal bovine serum (FBS), as expected^59,84^. Therefore, we repeated the same experiment using NAD^+^ and NMN precursors when comparing cells cultured in full media (FBS) or in serum free media (SFM). In addition, we shortened the time of incubation from 24 to 6 hrs in order to increase the chances to detect non-metabolized precursors by NMR analysis. We discovered that the distribution of metabolites when we compared SFM and FBS media supplemented with NMN and NAD^+^ was very different. Both NMN and NAD^+^ were stable in SFM but in FBS supplemented media, we identified NAM, NR, NMN and NAD^+^, suggesting degradation or breakdown of the metabolites in FBS-supplemented media (Fig. 6B).

Further, when we compared supernatants from MCF-7/Cas9 cells and MCF-7/CD73-KO cells in SFM and in FBS respectively, the profiles of precursor distribution looked similar. On the other hand, the distribution of NAD^+^ precursor between SFM and FBS among the same cell line looked very different. Specifically, NMN represented 100% of the profiles in SFM while in the presence of FBS supplemented media, the same precursor (NMN) was hydrolyzed to NR and NAM, with about 30–50% non-metabolized NMN detected. Similarly, the profiles of NAD^+^ supplementation varied between SFM and FBS supplemented media to the point that we were not able to detect NAD^+^ in the supernatants from cells cultured in FBS supplemented media. However, we identified that NAD^+^ was hydrolyzed to NMN, NR and NAM. Only a very small proportion of NAD^+^ was detected in Cas9 cells when incubated with NAD^+^ in the presence of FK866 (Fig. 6B). In the last set of experiments, we asked if heat inactivation of FBS would improve the stability of these small molecules. Testing heat inactivated serum (FBS-HI) and non-heat inactivated serum (FBS) revealed the presence of degraded metabolites of NAD^+^ and NMN was much more pronounced in FBS compared to FBS-HI supplemented media. Specifically, FBS-HI did not impact NMN while in FBS, NMN was hydrolyzed to NR and NAM. For NAD^+^ supplementation, although we did not detect un-metabolized NAD^+^ in the supernatants from FBS-HI containing media, we were able to show NMN and NR as major components. For cells cultured in FBS, NAD^+^ was degraded to NAM and a smaller proportion of NR. In all the conditions tested, the presence of FK866 (FK) did not provide additional changes (Fig. 6C).

### Impact of intracellular NAD^+^ content on base excision repair complex formation

**Figure 7:**
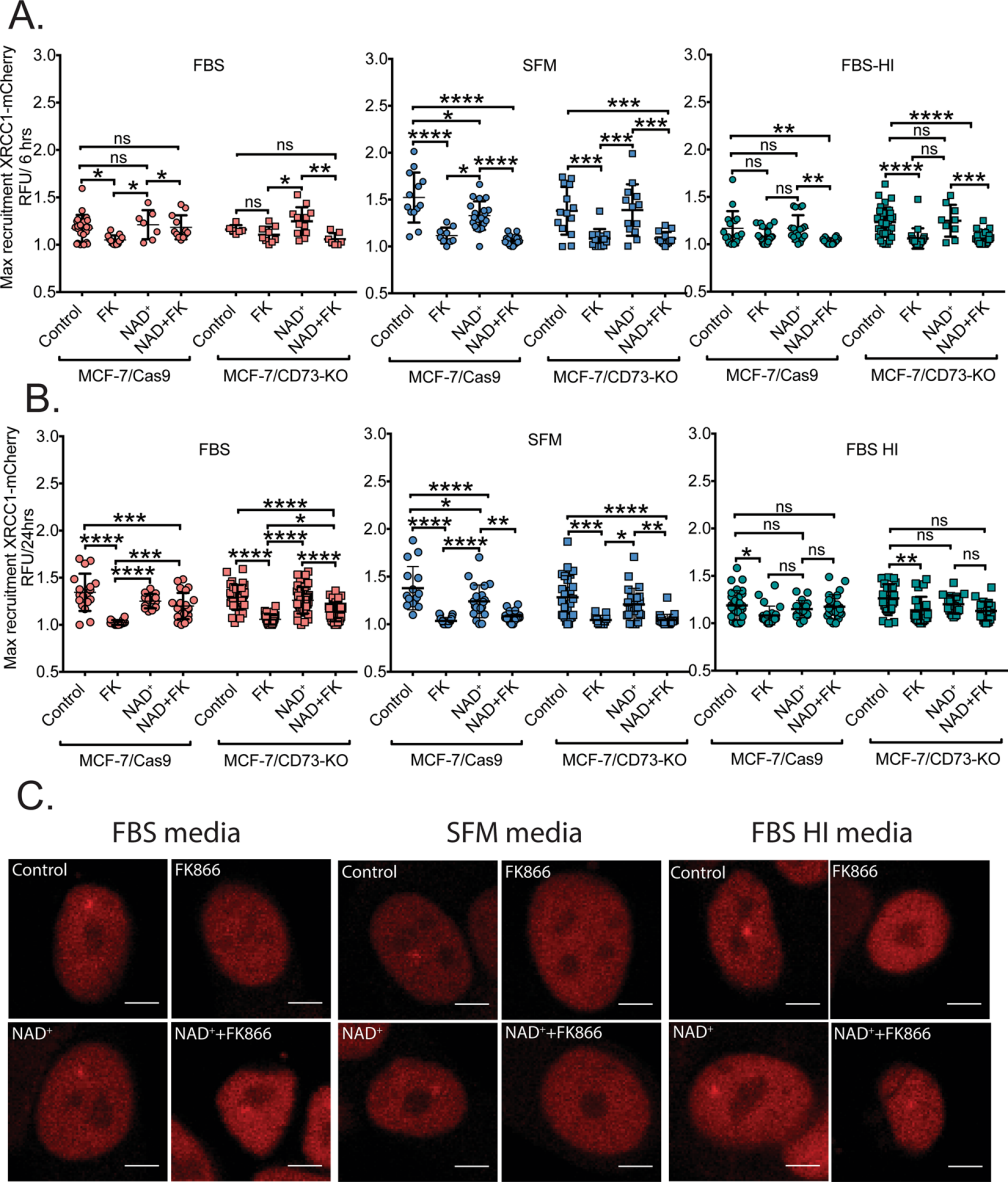
Impact of NAD^+^ depletion and NAD^+^ precursor supplementation on DNA repair complex formation. **(A, B)** Comparative measurements of maximal recruitment of XRCC1-mCherry protein to the site of laser-induced DNA damage: MCF-7/Cas9 vs MCF-7/CD73-KO cells exposed to NAD^+^ in the presence of serum-free media (SFM) or media supplemented with fetal bovine serum (FBS) or heat inactivated fetal bovine serum (FBS-HI) for 6 hrs **(A)** or 24 hrs **(B)**. **(C)** Representative images of maximal recruitment of XRCC1-mCherry in MCF-7/Cas9 cells cultured in media+FBS, SFM or media+FBS-HI; respectively, with differing NAD^+^ status.

As detailed above, NAD^+^ availability has a profound impact on genomic DNA damage levels and on DNA repair capacity in MCF-7 cells (Figs. 1 and S1). The most prominent DNA damage types that appear to be NAD^+^-dependent are those induced by alkylating (Fig. 1) and oxidizing agents, likely repaired by the base excision repair (BER) pathway^65^. Given the significant role that PARP1 plays in BER^15,16^, it is possible that suppressed NAD^+^ levels may negatively regulate the PARP1-dependent recruitment of the scaffold protein XRCC1^85^ to sites of DNA damage to form the BER complex. Therefore, we next asked if changes in intracellular NAD^+^ would affect the recruitment of DNA repair proteins to the site of damage and if the expression level of CD73 correlates with the outcome. To test this possibility, we expressed XRCC1 as a fusion with the fluorescent protein mCherry (XRCC1-mCherry) in the MCF-7/Cas9 and MCF-7/CD73-KO cells (Supplementary Fig. S7A) and utilized laser micro-irradiation confocal microscopy to induce DNA damage and quantify recruitment of the fluorescently labeled XRCC1 protein to sites of DNA damage. To induce DNA damage, we subjected cells to micro-irradiation using a laser with a 355 nm wavelength, which has been used to induce base lesions as well as DNA single-strand breaks (SSBs)^86^, as described in the methods section. We then measured BER/SSB repair complex assembly and disassembly (XRCC1 recruitment and resolution) at sites of DNA damage by measuring the mean intensity of fluorescence within a DNA damage region of the nucleus as compared to the mean fluorescent signal quantified for the entire nucleus, as described by Holton at al^87^. Before inducing damage, cells were depleted of NAD^+^ by treatment with FK866 (30 nM) and/or exposed to NAD^+^ precursors (NAD^+^, NMN and NR; 100 μM) for 6 or 24 hrs. We then evaluated the mean intensity of fluorescence of MCF-7/Cas9 and MCF-7/CD73-KO cells (Supplementary Fig. 6) as well as compared the maximum recruitment of XRCC1-mCherry between the two cell lines (Fig. 7).

We observed the same trend for XRCC1 recruitment in both the MCF-7/Cas9 and MCF-7/CD73-KO cell lines: rapid recruitment with a peak time of approximately 60 seconds and 50% resolution at approximately 200 seconds. As expected, recruitment was blocked when PARP1 was inhibited (Supplementary Fig. S7B,C). Further, NAD^+^ depletion (FK866 treatment) had a strong negative impact on XRCC1-mCherry recruitment to the site of DNA damage. This is consistent with the requirement for PARP1 activation for XRCC1 recruitment but may also imply other NAD^+^-dependent factors which may be at play, such as deacetylation by one of the SIRT family proteins^88–90^. In the absence of FK866 treatment, NAD^+^ or NAD^+^-precursor supplementation of the cells had little if any impact on DNA repair complex assembly and disassembly (Fig. 7A,B). This is in-line with the minor increase in cellular NAD^+^ levels seen when cells are supplemented with NAD^+^ or the precursors (Fig. 3C).

Further, we find that the FK866-effect can be reversed by supplementing cells with NAD^+^ (Figs. 7 and S6) as well as the NAD^+^ precursors NMN and NR (data not shown). However, NAD^+^ supplementation only reversed the recruitment defect in the FBS-containing media but not in SFM or FBS-HI-containing media (Figs. 7 and S6). In the FBS supplemented media, control cells, NAD^+^ or NAD^+^/FK866 supplemented cells exhibited robust XRCC1 recruitment with a complete lack of XRCC1 recruitment when NAD^+^ was depleted from the cells (Fig. 7). Conversely, in SFM and FBS-HI-containing media, NAD^+^ depletion inhibited XRCC1 recruitment but this could not be rescued by NAD^+^ supplementation (Fig. 7). Representative images of XRCC1 recruitment in FBS, SFM and FBS-HI, respectively at the maximum peak of the recruitment, are shown in Fig. 7C. Interestingly, in-line with our previous data (Fig. 5B), we observed a difference between 6 and 24 hr incubation with the precursors. In particular, for FBS and FBS-HI, an additional 18 hrs in the presence of NAD^+^ improved the recruitment of XRCC1-mCherry to the site of laser damage in cells treated with both NAD^+^ and FK866 (Fig. 7A,B), indicative of the role of the media in generating precursors of intracellular NAD^+^.

Collectively, our findings suggest that CD73 does not play a role in NAD^+^ precursor uptake and biosynthesis and importantly demonstrated the significance of intracellular levels of NAD^+^ on DNA repair complex formation, as measured by XRCC1 recruitment to sites of genomic DNA damage and the impact on the repair of alkylation-induced DNA damage.

## Discussion

NAD^+^ plays a significant role in cell metabolism not only as a cofactor for a majority of the redox reactions but also as a substrate for the consuming enzymes such as PARPs and Sirtuins^6,15,91^. Pathologic changes in NAD^+^ levels can therefore affect post-translational modification of proteins, the architecture of chromatin, DNA repair mechanisms and genomic stability^15,16^. One of the membrane-bound enzymes which has been suggested to participate in NAD^+^ precursor uptake and metabolism is CD73 (NT5E), an enzyme that is often upregulated in cancer cells^57,58,92,93^. Therefore, we decided to investigate the effect of alterations in NAD^+^ status on DNA damage and efficiency of DNA repair mechanisms including the CD73 enzyme as a target which could affect global NAD^+^ pools in cancer.

We tested this hypothesis first using our CometChip assay by exposing MCF-7 cells to different DNA damaging agents that allow us to demonstrate that NAD^+^ availability impacts DNA damage as well as DNA repair processes. We found that DNA damaging agents that require the base excision repair pathway (BER) to repair the DNA damage are highly dependent on the level of NAD^+^ (Figs. 1D and S1B). This is consistent with an earlier report by us evaluating the cellular response to the alkylating agent temozolomide^17^ and by others using a clinical model of genetic disease where the genomic defect caused hyperactivation of PARP and NAD^+^ deprivation^35^.

We next correlated these results with the expression of CD73. Since the commercially available CD73 enzymes did not show activity towards NAD^+^ or NMN (Supplementary Fig. S3A–D), we purified a soluble form of human, recombinant CD73 (rCD73) and also tested the CD73 ortholog from *Haemophilus influenza* (*Hi*NadN). Both the rCD73 and *Hi*NadN were highly active against AMP. However, rCD73 did not process NAD^+^ nor NADH whereas the *Hi*NadN enzyme was active against NAD^+^. However, neither enzyme could process NMN. From these analyses, we found no evidence for NAD^+^ hydrolysis catalyzed by the recombinant, purified human CD73 enzyme. Further, we find that the recombinant, purified human CD73 enzyme processes NMN extremely poorly, at least at physiologically relevant rates, as initially proposed^47,53^.

We next used four different mammalian cancer cell lines to test enzymatic properties of CD73 towards NAD^+^ and NMN metabolism. In-line with our biochemical analysis, the CD73 expression level did not correlate with intracellular NAD^+^ or NADH levels (Fig. 3C). It is noted that these data are in contrast with previously published analyses^53^, but the use of different cellular models and the preparation of FBS may explain the discrepancy. Since cells must sustain intracellular NAD^+^ homeostasis^94^, we used the NAMPT inhibitor FK866 to disturb the intracellular NAD^+^ balance and increase the cellular demand for NAD^+^ supplements (NAM, NR or NMN), where NAM and NR served as control supplements since neither one would need CD73 for uptake and/or metabolism^83^. The NMN supplementation for FK866 treated cells indeed increased the intracellular NAD^+^ content but there was no correlation between cellular CD73 expression and the ability to synthesize intracellular NAD^+^. In contrast, MCF-7 cells had the highest NAD^+^ content among the cell lines tested (Table 1 and Fig. 3A, B). MCF-7 cells utilize the NAD^+^ precursors very effectively as was also true for both the NR and NMN precursors (Fig. 3C**)**. Thus, we investigated if the MCF-7 cell line can be compensated by NRK1 enzymatic activity. NRK1 phosphorylates NR to NMN^50^ and could potentially explain the higher efficiency of MCF-7 in NAD^+^ precursor utilization (Fig. 4). However, the expression of NRK1 is not higher in MCF-7 cells as compared to the other cell lines (Fig. 4D) and our analyses were not impacted by treatment with the CD73 inhibitor, APCP (an analogue of ADP with well-established inhibition on CD73 enzymatic activity on AMP). Overall, these analyses further suggest that CD73 is not involved in extracellular NAD^+^- or NMN-bioprocessing.

In this study, the NAD^+^/NADH measurements were performed at 24 hrs post supplementation, an adequate time to deplete about 75% of the intracellular NAD^+^ level using FK866 (Figs. 1B and 4A–C) without affecting cell proliferation or viability^16,17^. Similarly, 24 hrs was sufficient for cells to take up the NAD^+^ precursors from the extracellular environment (media) and utilize each for NAD^+^ biosynthesis. However, it is important to consider changes in NAD^+^ metabolism, which could potentially take place rapidly upon supplement addition. To that end, we measured the level of the NAD^+^ precursors in cell supernatants by NMR analysis as well as the intracellular NAD^+^ levels in the presence of the NAD^+^ supplements and following treatment with FK866 (Fig. 5A, B). Analysis of supernatants suggested that the presence of serum in the media affected the stability of the precursors. In supernatants from MCF-7 cells, we also detected the presence of NAM, suggesting cells not only effectively utilize such ectoenzymes to metabolize NAD^+^ precursors but can actively perform NAD^+^ metabolite uptake (Fig. 5A). In parallel, when we measured NAD^+^ content in MCF-7 cells in a time dependent fashion, we observed that NR was able to restore NAD^+^ levels faster than NMN, peaking at 6 hrs post treatment (Fig. 5B). Based on these kinetic analyses, we concluded that 24 hrs of incubation is appropriate and all of the observed metabolite changes were increased in the presence of FK866.

Although the human soluble form of recombinant CD73 (rCD73) showed no activity for NAD^+^/NADH and very poorly for NMN, it is of value to determine the potential for CD73 to process NAD^+^ when comparing endogenously-expressed CD73 to the isogenic KO human cells. This point becomes of crucial interest in the tumor microenvironment in which both cancer and immune cells express a high level of CD73. To that end, we used the CRISPR/Cas9 editing technique to develop MCF-7/Cas9 and MCF-7/CD73-KO cells and measured intracellular NAD^+^ content and the impact by NAD^+^ or NAD^+^ precursor supplementation. Our NAD^+^ measurements do not support a role for CD73 activity towards any of the NAD^+^ precursors since the NAD^+^ content was the same for both cell lines **(**Fig. 6A**)**. We also took advantage of NMR analysis to verify the distribution of NAD^+^ precursors and their metabolites in cell supernatants. In-line with our biochemical analysis (Fig. 2B), the distribution of precursors for the MCF-7/Cas9 and MCF-7/CD73-KO cells were similar but the fact that we were able to detect NR when both cell lines were incubated with NMN indicated that an enzyme other than CD73 metabolizes NMN to NR. We also did not detect NAD^+^ in media supernatants from the samples treated with NAD^+^ but we were able to detect NMN, NR and NAM in these samples (Supplementary Fig. 5E). These data and the presence of NAM in the supernatants not exposed to cells (Fig. 5A) stimulated us to investigate if media by itself affects the stability of NAD^+^ precursors. Indeed, the presence of serum completely changed the distribution of the precursors (Fig. 6B). Metabolism of NAD^+^ precursors was observed regardless of using heat-inactivated serum or non-heat-inactivated serum although to a different degree and we observed intact NAD^+^ only in serum-free media (SFM) (Fig. 6B). These results suggest a very strong effect of serum on NAD^+^ precursor processing. Therefore, analysis of the SFM data is more informative than analysis of the distribution of the precursors present in the supernatants from FBS or FBS heat inactivated (FBS-HI) supplemented media. This experiment confirmed that neither the MCF-7/Cas9 nor MCF-7/CD73-KO cells have NMN-ectonuclotidase activity and are not able to metabolize NMN to NR. At the same time, both cell types showed some pyrophosphatase activity toward NAD^+^, which resulted in the accumulation of NMN and further hydrolysis to a small portion of NAM. This highlights the importance of using appropriately treated FBS in such studies (heat-inactivated) or to evaluate cells in SFM.

Next, we developed MCF-7/Cas9 and MCF-7/CD73-KO cells expressing XRCC1-mCherry (Supplementary Fig. S7A) in order to evaluate the impact of the intracellular levels of NAD^+^ on DNA repair complex formation. XRCC1 is a molecular scaffold protein and a critical component of the base excision repair (BER) and single-strand break repair (SSBR) machinery^65,95^. XRCC1 interacts with auto-modified PARP1 through its BRCT-I domain and thereby promotes XRCC1 recruitment to the site of DNA damage^96,97^. PARP1 is known to promote XRCC1 recruitment while PARP1 depletion or deletion has been shown to decrease XRCC1 accumulation to sites of UV laser induced DNA damage^98^. By using the laser micro-irradiation technique with a 355 nm wavelength, we were able to induce a focus of DNA damage in the cell nucleus, comprised mostly of base lesions and SSBs^86^, which triggered accumulation of XRCC1 to the site of damage. We demonstrated that in the presence of NAD^+^, PARP1 activation promotes XRCC1 recruitment and this recruitment or BER complex assembly was inhibited by NAD^+^ depletion mediated by treatment of the cells with the NAMPT inhibitor FK866 (Figs. 7A–C and S6). In-line with this observation, pre-treatment with the PARP1 inhibitor ABT-888 completely blocked the recruitment of XRCC1-mCherry, regardless of the media condition used (normal FBS, heat-inactivated FBS, and serum-free) (Supplementary Fig. S7B, C). Further, we also demonstrated that the expression status of the CD73 protein had no effect on the recruitment of XRCC1 since the dynamics of XRCC1-mCherry recruitment (assembly) and retention (disassembly) was the same in the MCF-7/Cas9 and MCF-7/CD73-KO cells (Figs. 7A–C and S6). The experiment in serum-free media (SFM) also showed similar results, where we did not observe an effect of the loss of CD73 on XRCC1 recruitment but we uncovered a strong effect of NAD^+^ content on DNA repair. Moreover, in SFM and in FBS-HI supplemented media, NAD^+^ supplementation was not able to compensate for FK866 inhibition and we did not observe XRCC1 recruitment to the site of damage in both cell lines treated with FK866 and NAD^+^ at 6 and 24 hrs (Figs. 7A,B and S6).

Collectively, in this study we were able to show that NAD^+^ availability impacted critical DNA repair processes that may play an important role in chemotherapeutic or genotoxic exposure, suggesting that NAD^+^ supplements should be considered in this context. In parallel, we documented that the CD73 ectoenzyme, although important for adenosine metabolism, does not impact NAD^+^ and/or NMN metabolism in the cell lines we tested. Interestingly, we also found, by NMR analysis, a strong effect of FBS on the stability of NAD^+^ precursors, which surprisingly has yet to be reported. In summary, after extensive analysis of several cancer cell lines as well as the isogenic MCF-7/Cas9 and MCF-7/CD73-KO cells, we saw no functional evidence for CD73 in NAD^+^ precursor uptake and biosynthesis. On the other hand, we documented a strong impact of NAD^+^ availability and intracellular NAD^+^ levels on DNA repair complex formation, as measured by XRCC1 recruitment to sites of genomic DNA damage as well as DNA damage caused by treatment with alkylating agents such as MMS and repair mediated by BER.

## Materials and Methods

### Chemicals and reagents

EMEM, alpha EMEM, RPMI1640, phosphate buffered saline (PBS), heat-inactivated fetal bovine serum (FBS-HI), L-glutamine, antibiotic/antimycotic, and geneticin were from ThermoFisher. Gentamycin was from Sigma (St. Louis, MO). FK866 (NIMH #F-901; IUPAC name: (E)-[4-(1-Benzyoylpiperidin-4-yl)butly]-3-(pyridin-3-yl)acrylamide; CAS number: 201034-75-5) was obtained from the National Institute of Mental Health Chemical Synthesis and Drug Supply Program (Bethesda, MD). FK866 was dissolved in DMSO to prepare a stock solution at a concentration of 1 mM and stored at −80 °C. Adenosine 5′-(α,β-methylene)diphosphate (APCP), an ADP analog, was purchased from Sigma (St. Louis, MO). APCP was dissolved in water at a concentration of 1 M and aliquots were stored at −20 °C. Nicotinamide adenine dinucleotide (NAD^+^) was purchased from Sigma (St. Louis, MO). Nicotinamide riboside (NR), nicotinamide (NAM), nicotinamide mononucleotide (NMN) were synthesized as described previously^16,51^. All NAD^+^ precursors were dissolved in water right before the experiments (NR) or dissolved and stored as aliquots at −80 °C (NAM, NMN, NAD^+^).

### CometChip analysis

The CometChip Platform is described in detail in our earlier report^62^. The CometChip Platform is comprised of disposable 30-micron CometChips (glass-backed CometChip cassettes with 1 mm agarose and micro-patterned micro-wells each at 30-micron width), a well-former that creates the 96-wells when assembled, the CometChip Electrophoresis System (CES) and the Comet Analysis Software (CAS), all described previously in detail^62^. These materials are available from BioTechne (Minneapolis, MN). Wild type MCF-7 cells or modified MCF-7/Cas9 or MCF-7/CD73-KO cells were plated in gelatin coated 96-well plates at a density of 2 × 10^5^ cells per well in culture media (100 μl). Some of the experiments required 24 hr pretreatment with the NAMPT inhibitor FK866 to efficiently deplete the intracellular NAD^+^ content. Treatments for DNA damage (1 hr) and DNA repair experiments were conducted in the 96-well plates with incubation taking place in a cell culture incubator at 37 °C. Compounds of interest (e.g., etoposide, MMS, rapamycin and others) or vehicle controls were diluted in full media and applied immediately to the cells in the 96-well plates. For the DNA repair experiments, the repair assay was initiated by removing the damaging agent, washing the cells with PBS (2×) and replacing fresh culture media in each well. Depending on the treatment, the kinetics of repair was different for each genotoxin (e.g., for 10 μM etoposide at 15, 30 and 60 min repair times allowed for complete repair of the accumulated damage). When the experimental procedure was finalized, cells were immediately harvested and loaded into the 30-micron CometChip. To allow proper loading of cells into the microwells under gravity force, the CometChip system was placed at 4 °C for 30 min. Next, the CometChip was washed twice with PBS and sealed with low melting point agarose (LMPA) (Topvision; ThermoFisher Scientific: 5-6 ml; 0.75% LMPA/PBS). The CometChip was then submerged in lysis solution with detergent (BioTechne) for 60 min at 4 °C. The CometChip was run under alkaline conditions (pH > 13; 200 mM NaOH, 1 mM EDTA, 0.1% Triton X-100). Electrophoresis was conducted at 22 V for 50 min at 4 °C. After the electrophoresis step, the CometChip was re-equilibrated to neutral pH using multiple washings with Tris buffer (0.4 M Tris·Cl, pH7.4 and 20 mM Tris·Cl, pH7.4). Subsequently, the DNA was stained with 1 × SYBR Gold dye (ThermoFisher Scientific) diluted in Tris buffer (20 mM Tris·Cl, pH7.4) for 30 min and de-stained for 1 hr in Tris buffer (20 mM Tris·Cl, pH7.4). Next, image acquisition was conducted on the Celigo S imaging cytometer (Nexcelom Bioscience; Lawrence, MA) at a resolution of 1 micron/pixel with whole plate imaging to avoid imaging variability. The Celigo S captures 16 images of each well that are then stitched into a single digital representation. Final image analysis was conducted using the dedicated comet analysis software (CAS) with the box size set to 220 × 161 pixels which represented a box size that would capture comets from heavily damaged cells without box overlap. Data acquired were exported to Excel (Microsoft) and subsequently to Prism 7 (GraphPad Prism) for data presentation and statistical analysis.

### ^1^H NMR analysis of the composition of NAD^+^/NAD^+^-metabolites in cell supernatants

#### Sample preparation for NMR analysis

One mL of each aqueous cell supernatant was freeze-dried and the residue obtained after drying was dissolved in 500 µL of D_2_O containing acetone (1 mM), used as the internal chemical shift reference (δH 2.17 ppm). Samples were vortexed three times and the clear homogeneous solutions were pipetted into a 5 mm NMR tubes for NMR analysis (^1^HNMR).

#### NMR Spectroscopy of cell supernatants

All one dimensional ^1^H NMR spectra were obtained at 300 K on a Bruker Ascend^TM^ 400 MHz ultrashielded spectrometer (Bruker Biospin) operating at 400.13 MHz for protons (9.39 Tesla). TopSpin 3.2 (Bruker BioSpin) was used for all NMR spectral acquisition (ns = 1024) and pre-processing and the automation of sample submission was performed using ICON-NMR (Bruker BioSpin). All samples were automatically shimmed and their acquisition time was 1 hr 18 minutes.

#### ^1^H NMR and data collections

The sensitivity of the 400 MHz instrument is low compared to NMR instruments currently used for similar experiments. However, the increased number of scans, the use of an internal standard, manual peak peaking and manual measurement of the individual peak intensity aimed to compensate to a large extent for this limitation. Upon acquisition of the ^1^H-MR spectra and referencing to the acetone peak with a relative intensity of 6 (6 equivalent H/molecule of acetone), peaks which corresponded to NAM, NR, NMN and NAD^+^ were manually identified and the relative intensity of these peaks to that of acetone were recorded. No buffer was used for spectrum acquisition as the changes in the chemical shifts were unnoticed over the course of the incubation period even as the media’s pH might have changed. As such, samples were directly measured for their relative chemical composition. Because each of these metabolites have multiple hydrogens with specific chemical shifts, relative intensities for each composition could be validated internally. Changes in the intensity of the ^1^Hs of NAD^+^ and each of its derivatives relative to the acetone standard could thus be measured and reported for each sample as a % fraction relative to the total NAD^+^ and NAD^+^-metabolite composition. Comparison between samples provided information as to the relative changes of % composition of NAD^+^ and NAD^+^-metabolites in the supernatants.

### Cell culture

All cells were cultured at 5% CO_2_ and 37 °C. LN428 glioblastoma cells and derived cell lines were cultured in alpha EMEM supplemented with 10% heat inactivated FBS (FBS-HI), L-glutamine, antibiotic/antimytotic and gentamycin^99^ (see Supplementary Table S1 for cell line details). LN428 is an established glioblastoma-derived cell line with mutations in p53, deletions in p14^ARF^ and p16 and is WT for PTEN^100,101^. The T98G glioblastoma cell line was purchased from American Type Culture Collection (ATCC) and cultured in EMEM supplemented with 10% heat inactivated FBS (FBS-HI), non-essential amino acids, sodium pyruvate and antibiotic/antimytotic^102^. The MDA-MB-231 breast cancer cell line is a generous gift from Dr. Julie Eiseman (University of Pittsburgh) and cultured in RPMI 1640 supplemented with 10% FBS and gentamycin. The MCF-7 breast cancer cell line was purchased from American Type Culture Collection (ATCC) and cultured in EMEM supplemented with sodium pyruvate, insulin, non-essential amino acids and 10% FBS, FBS-HI or in the absence of serum (SFM) as indicated in the text. The BT-483, BT-549, HCC-1395, MCA-MB-157, MDA-MB-361, BT-20 and HCC-38 breast cancer cells were kindly provided by D. Lansing Taylor (University of Pittsburgh). All cell lines are periodically tested for mycoplasma contamination (and shown to be negative) and in addition they were tested for cell line authentication by Genetica DNA Laboratories - a LabCorp brand – analysis is included in the Supplementary Materials (Supplementary Table S2).

### Development of MCF-7/CD73-KO and MCF-7/Cas9 cells

We developed MCF-7 cell lines with a stable knockout of CD73 (NT5E) using the two vector CRISPR/Cas9 system (plentiGuide-Puro together with plenticas9-Blast to deliver hSpcas9 and blasticidin resistance to cells)^81,82^. Both vectors were from Addgene (plasmid #52963 and #52962). To perform the knockout of CD73, we designed the guide RNA (gRNA) using the ChopChop software package (http://chopchop.cbu.uib.no). The resulting gRNA (CD73-5’ TAGTCACTTCTGATGATGGG) was predicted to cut at genomic location chr6:85485280 in exon 4 of the NT5E gene. Control cell lines expressing Cas9 were created using Addgene plasmid #52962 without a gRNA insert. Cells were maintained in media containing the puromycin selection agent (1 μg/ml) for 12 days before protein immunoblot validation of the knockout was performed. Details of the technique have been described in Sanjana *et al*.^81^.

### Expression, purification and activity analysis of human CD73

Nucleotidase activity assays were performed with purified recombinant enzyme by quantifying the inorganic phosphate produced from CD73-catalyzed AMP hydrolysis. Briefly, recombinant CD73 (soluble form, residues 27-549 including a His-tag at the C-terminus) was produced in Sf9 insect cells using the pFastBac baculovirus system (ThermoFisher Scientific) and purified by two consecutive steps (Ni-MTA affinity and size exclusion chromatography) as previously described^78^. To determine the enzymatic activity, recombinant CD73 (2 nM, final concentration) was incubated in a reaction buffer (Tris 50 mM, pH7.5, NaCl 100 mM, MgCl_2_ 1 mM, CaCl_2_ 1 mM and ZnCl_2_ 5 µM) and reaction was started by addition of either the natural substrate (AMP) or the test substrates NAD^+^, NADH or NMN (nicotinamide mononucleotide), with concentrations ranging from 0 to 250 µM, and incubated for 2.5 minutes at 37 °C under gentle shaking. Then, the reaction was stopped by addition of the Green Malachite reagent (Phosphate assay kit, Sigma) and production of inorganic phosphate was quantified by reading the optical density at 630 nm on a plate reader (Tecan Sunrise). A standard phosphate concentration range (0–50 µM) for normalizing raw data was included and the results correspond to the average of three independent experiments (GraphPad Prism 8 was used for analyzing and plotting the data).

### DNA Microarray analysis

Comparative analysis of mRNA expression was performed using the Human U219 Array Strip and the Affymetrix GeneAtlas system, as per the manufacturer’s instructions. Microarray analysis for each of the cell cultures (in triplicate) was accomplished with 100 ng purified total RNA as the initial material and the corresponding amplified and labeled antisense RNA (aRNA) using an GeneChip 3′ IVT Express kit (Affymetrix), as described by the manufacturer. The resulting aRNA was fragmented as described by the manufacturer. The labeled aRNAs were then mixed with hybridization master mix, and the hybridization cocktails were then denatured at 95 °C for 5 min, followed by 45 °C for 5 min, and then kept at 45 °C until applied to the hybridization tray (GeneAtlas System; 120 μL hybridization cocktail of a cell culture was transferred into a well of a four-well hybridization tray). The array strip was immersed into hybridization mixture and incubated in the hybridization station at 45 °C for 16 h. After hybridization, the strip was washed and stained in the GeneAtlas Fluidics Station using the GeneAtlas Hybridization, Wash, and Stain Kit (#900720; Affymetrix), and the intensity of each hybridized probe was generated using the GeneAtlas Imaging Station. Raw.cel files from the Human U219 Array Strip were analyzed using the “affy” package in R Bioconductor. The raw data were normalized and summarized using the robust multichip average method (RMA). At this point, each gene is represented by one or more probe sets. The probe sets expressing <75 units for all samples were filtered out for the genes that have other probe sets that are being expressed (>75 units). The selective filtering was performed to avoid getting rid of any gene altogether. For genes represented by multiple probe sets, the probe set with the highest interquartile range (a descriptive statistic used to summarize the extent of the spread of the data) was selected to represent the gene. As a result of the filtering procedure, all genes are represented by a single probe set for further statistical analysis.

### NAD^+^ measurements

The level of NAD^+^ and NADH in cells was measured using the Enzychrome NAD^+^/NADH colorimetric assay kit (BioAssay Systems) as we have described previously^16,103^. Briefly, cells were seeded in a 6 well plate at the density 2 × 10^5^ cells per well for NAD^+^ measurements and 3 × 10^5^ cells per well for NAD^+^ pool measurements (NAD^+^ and NADH). 24 hrs later, cells were treated with the indicated NAD^+^ precursors (100 μM) in the presence or absence of FK866 (30 nM). Following 24 hrs of treatment, cells were harvested and a suspension of 2 × 10^5^ cells was divided in half for measuring NAD^+^ and NADH respectively or a suspension of 1 × 10^5^ cells was used for the NAD^+^ measurement only. Cell pellets were homogenized using plastic pestles and the extraction of NAD^+^ and NADH was performed in the provided lysis buffers. Extracts were heated at 60 °C for 5 min and neutralized with the provided buffers. Samples were spun down and the supernatant was immediately used for measurements of NAD^+^/NADH content using a Molecular Devices VersaMax™ tuneable plate reader at 565 nm.

### Cell extract preparation and immunoblot

Cells were seeded at 4 × 10^5^ cells per 60 mm dish, 24 hrs prior to treatment. For the experiments to evaluate the endogenous level of proteins as well as changes in protein expression following knockout: after 24 hrs, cells were washed twice with cold PBS and whole cell extracts were prepared using 2X clear Laemmli buffer (2% SDS, 20% glycerol and 63 mM Tris-HCl pH6.8, in the absence of bromophenol blue). For the time course experiments: cells were seeded at 2 × 10^5^ cells per well in 6-well plates 24 hrs before the treatment. Next, cells were treated with FK866 (30 nM) for 0.5, 1, 6, 16 or 24 hrs. At the end of the treatment period, cells were washed twice with cold PBS and whole cell extracts were harvested as described above. Protein concentrations were determined using the DC protein assay (Bio-Rad) according to the manufacturer’s instruction. Thirty micrograms of protein were loaded on precast 4%-12% Tris-Bis Novex® NuPAGE® SDS-PAGE gels (Life Technologies) and samples were subjected to electrophoresis (100 mV for 2 hrs). For transfer, we used the Turbotransfer semi-dry system (BioRad). The primary antibodies utilized in the course of the study were an anti-CD73/NT5E rabbit monoclonal Ab (13160S, Cell Signaling Technology, diluted in 1% milk in TBST at 1:1000), an anti-NRK1/C9orf95 [EPR11190] rabbit monoclonal Ab (ab169548; Abcam, diluted in in 1% milk in TBST at 1:5000), an anti-CD38 mouse monoclonal Ab (Cat#611114, BD Transduction Laboratories; diluted in 1% milk in TBST at 1:1000), an anti-XRCC1 rabbit polyclonal Ab (A300065A, Bethyl; diluted in 1% milk in TBST at 1:1000) and the anti-CRISPR-Cas9 mouse monoclonal Ab (7A9–3A3, Novus, diluted in 1% milk in TBST at 1:1000). The anti-β-actin mouse monoclonal Ab (sc-56, Santa Cruz; diluted in 1% milk in TBST at 1:1000) and the anti-PCNA (PC10) mouse monoclonal Ab (sc-56, Santa Cruz; diluted in 1% milk in TBST at 1:1000) were used to evaluate actin or PCNA protein levels as a loading control. Image Lab (BioRad) was utilized both to image and to analyze densitometry of the immunoblot images. Secondary antibodies included the Goat anti-Mouse IgG H+L-HRP conjugate (Cat#170-6516, Bio-Rad; dilution 1:2500) and the Goat anti-Rabbit IgG H+L-HRP conjugate (Cat#170-6515, Bio-Rad; 1:2500).

### Quantitative RT-PCR analysis

Expression of CD73/NTE5 and SLC12A8 mRNA was measured by quantitative RT-PCR (qRT-PCR) using an Applied Biosystems StepOnePlus system, as we have described previously^103^. Briefly, 80,000 cells were lysed and reverse transcribed using the Applied Biosystems Taqman® Gene Expression Cells-to-CT kit, as described previously^104^. Analysis of mRNA expression was performed as per the instruction of the manufacturer, via the ∆∆CT method. We used the Applied Biosystems TaqMan Gene Expression Assay probe for human CD73 (Hs00296200_m1) or for SLC12A8 (Hs00226405_m1) and then each was normalized to the expression of human β-actin (Hs99999903_m1). Samples were run in triplicate and the results shown are the mean ±SD of all three analyses.

### Lentiviral expression of XRCC1-mCherry in MCF-7/Cas9 and MCF-7/CD73-KO cells

A lentiviral vector was designed for expression of human XRCC1 as a C-terminal fusion with the fluorescent protein mCherry (pLV-CMV-hXRCC1-mCherry-Hygro; VectorBuilder) and expressing the hygromycin resistance gene to allow selection (see Supplementary Table S3). Lentiviral particles were generated by co-transfection of 4 plasmids [Control plasmid (pLVX-IRES-Puro) or the plasmid expressing XRCC1 (pLV-CMV-hXRCC1-mCherry-Hygro), together with pMD2.g (VSVG), pVSV-REV and pMDLg/pRRE into 293FT cells using TransIT®-2020 Transfection reagent. The collection and isolation of lentiviral particles and transduction of MCF-7 cells were performed as we have described previously^16,99^. Stable cell lines were developed by selection in hygromycin (200 μg/ml) for 2 weeks. Expression of XRCC1-mCherry was evaluated by immunofluorescence (IF) to confirm nuclear localization (Supplementary Fig. S7A) and by immunoblot to confirm molecular weight and expression of the complete fusion protein (not shown). Immunoblot conditions for XRCC1 are described above.

### Laser micro-irradiation

1 × 10^5^ MCF-7/Cas9/XRCC1-mCherry and MCF-7/CD73-KO/XRCC1-mCherry cells were plated onto 4-well cover-glass bottom chamber slides (Lab-Tek II, Thermo Fisher Scientific). 24 hrs later, cells were subjected to treatment with NAD^+^ or NAD^+^-precursors (NMN, NR, NAM) and/or the NAMPT inhibitor FK866. Alternatively, 24 hrs later, cells were subjected to treatment with the PARP1 inhibitor ABT-888 (10 μM, 30 min). Depending on the experimental design, cell treatment was performed in FBS supplemented media, FBS-HI supplemented media or in serum-free media (SFM). Following the 24 hr treatment, a 3 × 3 pixel region of interest (ROI) within the nucleus of selected cells was irradiated with a 355 nm laser (PicoQuant) for 1.0 second using a 40x oil immersion objective (NA 1.3). Pre-irradiation and post-irradiation sequential images were taken for 10 minutes at 15 second intervals. For the XRCC1-mCherry recruitment experiments, the maximum value of intensity was selected from 3–4 independent experiments where 5–10 individual cells were analyzed for each experiment. Data are presented as a scatter dot plot. Micro-irradiation and fluorescence imaging were performed with a Nikon A1rsi laser scanning confocal microscope (Nikon Instruments), and analysis was performed with FIJI.

### Statistical analysis

All data is shown as a mean +/− standard error from 3 or 4 independent experiments. Student’s t-test was used for comparisons between two groups. For multiple comparisons, one or two-way ANOVA followed by post-hoc Tukey’s multiple comparison test was used. Statistical analysis was performed using GraphPad PRISM 7 or 8, as indicated.

## Supplementary information

Supplementary Information.
