# Supplementary material for: Extracellular NAD+ enhances PARP-dependent DNA repair capacity independently of CD73 activity
Source: Sci Rep. 2020 Jan 20;10:651. doi: 10.1038/s41598-020-57506-9 (PMC6971268; doi:10.1038/s41598-020-57506-9)
Supplement: Supplementary file 1 — Supplementary Information. [file 41598_2020_57506_MOESM1_ESM.pdf]

Supplementary Information for:

Title

Extracellular NAD$^+$ enhances PARP-dependent DNA repair capacity independently of CD73 activity

Running Title

CD73-independent DNA repair

Authors

Anna Wilk[1,2], Faisal Hayat[1,2], Richard Cunningham[1,2], Jianfeng Li[1,2], Silvia Garavaglia[3], Leila Zamani[1], Davide M Ferraris[3], Peter Sykora[1,#], Joel Andrews[1], Jennifer Clark[1,2], Amanda Davis[1], Laurent Chaloin[4], Menico Rizzi[3], Marie Migaud[1,2] & Robert W. Sobol[1,2]*

Affiliations

[1]Mitchell Cancer Institute, University of South Alabama, Mobile, AL 36604, USA

[2]Department of Pharmacology, College of Medicine, University of South Alabama, Mobile, AL 36604, USA

[3]Department of Pharmaceutical Sciences, University of Piemonte Orientale, Largo Donegani 2, 28100 Novara, Italy

[4]Institut de Recherche en Infectiologie de Montpellier (IRIM), Université de Montpellier, CNRS, 34293 Montpellier, France

[#]Current address: Amelia Technologies; 14676 Rothgeb Drive; Rockville, MD, 20850

***Corresponding author:** Robert W. Sobol, Ph.D.
Mitchell Cancer Institute
Department of Pharmacology
University of South Alabama
1660 Springhill Avenue
Mobile, AL 36604
Tel: (251) 445-9846
rwsobol@health.southalabama.edu

Supplementary Methods

Cell Line Authentication. LN429, T98G, MDA-MB-231 and MCF-7 cell lines were prepared and sent for authentication to Genetica DNA Laboratories - a LabCorp brand. Briefly, 75% confluent dishes of each of the tested cells were washed with PBS and trypsynized in order to prepare single cell suspensions. Next, cells were spun down and subsequently the pellets were washed twice in PBS to completely removed traces of trypsin. Cell pellets in 100 µl PBS were snap-frozen and shipped to Genetica on dry ice. A Short Tandem Repeat (STR, PowerPlex16HS – HUMAN specific; includes a mouse marker for detection of mouse DNA) comparative analysis was performed and a Database Profile together with peak data were obtained (see **Supplementary Table S2**).

Expression and purification of *Hi*NadN. *Escherichia coli* BL21(DE3) cells (Stratagene) were transformed with the expression vector for *Hi*NadN (pET25b-*Hi*NadN), the bacteria were inoculated in 1 liter of 2XTY medium supplemented with 50 µg/ml ampicillin and incubated for 4 h at 30°C under vigorous shaking. The absorbance was constantly monitored until reaching a value of 0.6, the growing cells were then supplemented with 0.3mM IPTG (isopropyl β-D-thiogalactopyranoside) to induce gene expression, and the bacterial culture was further incubated for 3 h at 30°C. The bacterial cells were then collected by centrifugation, washed 1X with PBS and frozen at -20°C. To purify the enzyme, the bacterial pellet from 1 liter of *Hi*NadN expressing culture was resuspended in 50 ml of lysis buffer [10mM KH_2PO_4 pH6.8, 1000 units of Benzonase® nuclease and an EDTA-free protease inhibitor cocktail], subjected to two cycles at 1.6 kBar in a refrigerated mechanical disruption system (Basic Z apparatus, Constant Systems). Next, the pellet and supernatant were separated by centrifugation. $(NH_4)_2SO_4$ was added to the clarified lysate to 30% saturation and the resulting precipitate was removed by centrifugation at 10,000xg for 30 min at 4°C. The soluble fraction was dialyzed overnight at 4°C against a solution containing 10 mM KH_2PO_4, pH 6.8 and loaded onto a ceramic hydroxyapatite column (CHP) equilibrated with the same solution; elution of the proteins was performed by applying a linear gradient from 10 to 500mM of a KH_2PO_4 buffer pH6.8. Pure fractions, as determined by standard SDS/PAGE analysis, were pooled, dialyzed against a solution containing 5mM KH_2PO_4 pH 6.8, 100mM Tris/HCl pH8.5 and 10mM $MgCl_2$, and

concentrated by ultrafiltration using a 30000 MWCO (molecular weight cut-off) disposable Vivaspin device (Vivascience) to a final protein concentration of 10 mg/ml, as determined by a Bradford assay (Sigma). Aliquots of highly homogeneous *Hi*NadN were used for enzymatic investigations and stored at -20°C.

^{31}P *Hi*NaDN Assay. Purified, recombinant *Hi*NadN (10.5 µg) was added to a freshly prepared 5mM solution of NAD^+ in buffer (100mM Tris buffer/HCl pH8.5; 10 mmol $MgCl_2$; 400µl), diluted to a final volume of 500µl with additional buffer. The sample spectra were performed using decoupled ^{31}P NMR on a Bruker 400 MHz spectrometer with 126 scans at ambient room temperature.

Laser micro-irradiation: recruitment of XRCC1 and modulation by PARP1 inhibition. $1x10^5$ MCF-7/Cas9/XRCC1-mCherry and MCF-7/CD73-KO/XRCC1-mCherry cells were plated onto 4 well cover-glass bottom chamber slides (Lab-Tek II, Thermo Fisher Scientific). 24 hrs later, cells were subjected to treatment with the PARP inhibitor ABT-888 (10 µM, 30 min). A 3x3 pixel region of interest (ROI) within the nucleus of selected cells was the irradiated with a 355nm laser (PicoQuant) for 1.0 second using a 40x oil immersion objective (NA 1.3). Pre-irradiation and post-irradiation sequential images were taken for 10 minutes at 15 second intervals. For the XRCC1-mCherry recruitment experiments, the maximum value of intensity was selected from 3-4 independent experiments where 5-10 individual cells were analyzed for each experiment. Data are presented as a bargraph plot. Micro-irradiation and fluorescence imaging were performed with a Nikon A1rsi laser scanning confocal microscope (Nikon Instruments), and analysis was performed with FIJI.

Supplementary Results

Analysis of NAD$^+$ catabolic activity of commercial preparations of CD73 and of *Hi*NadN. We have evaluated commercially available mammalian CD73 protein preparations using phosphorus (^{31}P) NMR in order to test their activity towards NAD$^+$, NMN and AMP. Unfortunately, each enzyme preparation was devoid of any enzymatic activity for all substrates, as demonstrated in **Supplementary Fig. S3A-D**. In addition, we evaluated the enzymatic activity of *Hi*NadN, the *Haemophilus influenza* orthologue of the human CD73[1], by phosphorus NMR (^{31}P-NMR). It is important to emphasize that ^{31}P-NMR analysis provides accurate quantification of pyrophosphatase and C-5 nucleotidase enzymatic activities of *Hi*NadN on NAD$^+$, to its hydrolytic products: adenosine monophosphate (AMP), β-nictotinamide mononucleotide (NMN) and inorganic phosphate without additional sample manipulation. Based on the study by Garavaglia *et al.*[1], it was anticipated that NAD$^+$ would be cleaved to AMP and NMN through the pyrophosphatase activity of *Hi*NadN, then further to nicotinamide riboside (NR), adenosine and inorganic phosphate via C-5 nucleotidase activity as outlined in **Fig. 2A**. However, such progression of events was not observed. At 240 min after the start of the reaction, NAD$^+$ was completely consumed but the final reaction products included both NMN (3.4 ppm) and phosphate (2.4 ppm), at matching intensity of integrals (**Supplementary Fig. S4A & S4B**). As such, it could be deduced that AMP had been completely consumed to form adenosine and inorganic phosphate, whereas, NMN had been left untouched by the C-5 nucleotidase activity of the *Hi*NadN enzyme (**Supplementary Fig. S4B**). Additional spiking of the ^{31}P-NMR sample with an excess of pure NMN further confirmed the identity of the peak to be NMN, at 3.4 ppm (**Supplementary Fig. S4C**). The same ^{31}P-NMR experiment was repeated using *Hi*NadN in the presence of either NMN or AMP. After 12 h (or even longer) incubation at 37˚C, no hydrolysis (C-5 nucleotidase activity) could be observed for NMN, whereas AMP was rapidly consumed to form inorganic phosphate and adenosine (**Supplementary Fig. S4D**). These results suggest that NMN is not a substrate for the enzyme *Hi*NadN and given that it is an orthologue of the human CD73, one can predict that NMN is not likely a substrate for the human enzyme.

Laser micro-irradiation: recruitment of XRCC1 and modulation by PARP1 inhibition. MCF-7/CD73-KO and the control MCF-7/Cas9 expressing cells were cultured in different media conditions (normal FBS, heat-inactivated FBS, and serum-free) and treated with the PARP1 inhibitor ABT888. Cells were irradiated with a 355nm laser and recruitment of XRCC1-mCherry was analyzed. Relative recruitment intensities for each damaged cell were calculated (max fluorescence intensity/pre-damage fluorescence intensity), and compared for each cell line by Two-Way ANOVA (inhibitor treatment was the row factor, and media condition was the column factor). Here we show that media condition does not modify the effect of PARP1 inhibition on recruitment and that PARP1 inhibition completely blocks XRCC1-mCherry recruitment to the site of laser-induced DNA damage (**Supplementary Fig. S7B,C**).

Supplementary Figure Legends

Supplementary Figure S1. DNA damage and repair measured by CometChip assay to validate the effect of NAD^+ depletion. (A) CometChip results showing all points and spread of data for DNA damage and repair in MCF-7 cells exposed for one hr to etoposide (10μM) in the presence or absence of FK866. The time points of repair are indicated at the bottom. Plots are the average of 8-12 wells from two or three independent CometChips; 300-1500 comets per treatment. Statistical analysis was performed using GraphPad Prism 7 and one-way ANOVA followed by *post-hoc* Sidak's multiple comparison test (p=ns<0.3656). **(B)** Dose-response analysis of MMS treatment shows dose-dependent increase in damage accumulation (black and red) and slower repair mechanisms when NAD^+ was depleted by FK866 treatment (grey and teal). Data are expressed as % Tail DNA with error bars representing mean +/− 95% CI. Plots are the average of 8 wells from two independent CometChips; >1000 comets per treatment. Statistical analysis was performed using GraphPad Prism 7 and one-way ANOVA followed by *post-hoc* Sidak's multiple comparison test (ns= not significant, **p=0.0076, ****p<0.0001).

Supplementary Figure S2. Integrated genomic analysis using the cBio portal to evaluate genes for NAD metabolism and NAD consuming enzymes and CD38 or PNP expression analysis. An analysis[2,3] of mutations, amplifications and deep deletions for the **(A)** genes for NAD^+ *de novo* pathway enzymes, **(B)** genes for NAD^+ salvage pathway enzymes and **(C)** genes for NAD^+-consuming enzymes, ranked by cancer type, comparing breast cancer and gliomas. **(D)** Immunoblot analysis of the expression of CD38 in Raji cells (positive control) and the 4 cell lines used in this study: LN428, T98G, MCF-7 and MDA-MB-231. PCNA was used as a loading control. **(E)** Relative mRNA levels of CD38 in the fibroblast cells GM09607 (positive control) and the 4 cell lines used in this study: LN428, T98G, MCF-7 and MDA-MB-231. **(F)** Immunoblot analysis of the expression of NRK1 in nine breast cancer cell lines. PCNA was used as a loading control (same cell lysates as in **Figure 3B** and same loading control as used in **Figure 3B**). **(G)** Correlation of PNP mRNA expression and NADH levels in Control and NR supplemented cells (LN428, MDA-MB-231, MCF-7 and T98G) as measured in **Fig. 2A**: <u>left plot</u>: Control; <u>center plot</u>: NR treatment; <u>right plot</u>: NR vs Control.

Supplementary Figure S3. NMR analysis of the enzymatic activity of commercial preparations of human CD73 on NAD^+. (A) ^{31}P NMR study showing the NAD^+ peak at its corresponding ppm over time. The peak at 0.3 ppm likely comes from the phosphate in the enzyme solution which reaches its maximum intensity without any further increase observed. **(B)** ^{31}P NMR assay spiked with an excess of pure NMN, confirming a lack enzymatic activity of the CD73 enzyme towards NAD^+ and NMN; the presence of an additional peak at 0.3 ppm was observed. **(C)** ^{31}P NMR assay spiked with an excess of pure AMP, confirming a lack of enzymatic activity of the CD73 enzyme towards AMP similarly to other substrates such as visible NAD^+ and NMN; the presence of an additional peak at 0.3 ppm was observed. **(D)** ^{31}P NMR study showing a lack of enzymatic activity of the CD73 enzyme over time (2, 5 and 16 hours). A Lack of a change in the ratio of AMP/NMN to phosphate integrals was observed. **(E)** Immunoblot analysis of NRK1 expression in the four cancer cell lines LN428, MDA-MB-231, MCF-7 and T98G. Actin was used as a loading control (independent replicates of results demonstrated in **Fig. 4D**).

Supplementary Figure S4. NMR analysis to evaluate the phosphatase activity of *Hi*NadN on NAD^+. (A) ^{31}P NMR study indicating the conversion of NAD^+ to NMN and inorganic phosphate, over a time course of 240 min; arrows indicate each breakdown product and their corresponding ppm. **(B)** Integration of peaks at t = 240 min, matching intensity of integrals of the final degradation products shows that the quantity of remaining NMN is equivalent to that of inorganic phosphate after all the AMP and NAD^+ has been consumed. **(C)** ^{31}P NMR assay spiked with an excess of pure NMN after complete conversion of both AMP and NAD^+, confirming the peak identity of NMN at 3.4 ppm. **(D)** ^{31}P NMR assay repeated with only NMN as the substrate for the *Hi*NadN enzyme shows no C-5 nucleotidase activity.

Supplementary Figure S5. Comparative analysis of the effect of CD73 inhibitor (APCP) on NAD^+ biosynthesis in cancer cell lines. Measurement of intracellular NAD^+ content in glioblastoma cell lines **(A)** LN428 and **(B)** T98G as well as two breast cancer cell lines **(C)** MDA-MB-231 and **(D)** MCF-7 in the presence of APCP and FK866 inhibitors and NMN supplementation. Histograms are the average of two or three independent NAD^+ measurement assays. Statistical analysis was performed using GraphPad Prism 7 and one-way ANOVA followed by *post-hoc* Tukey's multiple comparison test (p=ns). **(E)** NMR analysis of the distribution of MCF-7/Cas9 and MCF-7/CD73-KO cell supernatants exposed to NAM, NR, NMN and NAD^+ in the presence and absence of FK866 for a period of 24 hrs. During the experiment, cells were cultured in the media containing fetal bovine serum (non-heat inactivated). **(E)** Measurement of mRNA expression for Slc12a8 in the 4 cell lines used in this study: LN428, T98G, MCF-7 and MDA-MB-231; as determined by qRT-PCR analysis and normalized to the expression of human β-actin mRNA via the $\Delta\Delta$CT method.

Supplementary Figure S6. Impact of NAD^+ depletion and supplementation on DNA repair complex formation. Dynamics of recruitment of XRCC1-mCherry to the site of laser-induced DNA damage in MCF-7/Cas9 and MCF-7/CD73-KO cells supplemented with NAD^+ in the presence of FBS (fetal bovine serum), SFM (serum free media) and FBS-HI (heat inactivated fetal bovine serum) exposed to NAD^+ supplementation for 6 hrs **(A)** or 24 hrs **(B).**

Supplementary Figure S7. Nuclear localization and characterization of the transgene XRCC1-mCherry and PARP1 inhibitor impact on recruitment. (A) Confocal image demonstrating nuclear localization of the XRCC1-mCherry transgene expressed in MCF-7 cells. **(B, C)** Comparative measurements of maximal recruitment of XRCC1-mCherry protein to the site of laser-induced DNA damage: **(B)** MCF-7/Cas9 or **(C)** MCF-7/CD73-KO cells were cultured in the presence of serum-free media (SFM) or media supplemented with fetal bovine serum (FBS) or heat inactivated fetal bovine serum (FBS-HI) for 24 hrs and then exposed to the PARP1-inhibitor ABT-888 (10 μM, 30 min) prior to laser-induced DNA damage induction.

Supplementary Data – Full Blot Images

As per journal policy, all gels/blots as shown are from the same gel. If cropped from different parts of the same gel, or from different gels, fields, or exposures then that is indicated. In the figures, cropped gels/blots are displayed. Full-length gels and blots are shown here for the indicated figures.

Supplementary Tables

Supplementary Table S1. Cell Lines. Human cell lines developed and used in this study.

Supplementary Table S2. Cell Line Authentication. A Short Tandem Repeat (STR) comparative analysis was performed by Genetica/LabCorp (PowerPlex16HS – HUMAN specific; includes a mouse marker for detection of mouse DNA).

Supplementary Table S3. Vectors / plasmids. Vectors developed for and used in this study.

References cited in the Supplementary Information

1 Garavaglia, S. *et al.* The high-resolution crystal structure of periplasmic Haemophilus influenzae NAD nucleotidase reveals a novel enzymatic function of human CD73 related to NAD metabolism. *Biochem J* **441**, 131-141, doi:10.1042/BJ20111263 (2012).

2 Gao, J. *et al.* Integrative analysis of complex cancer genomics and clinical profiles using the cBioPortal. *Sci Signal* **6**, pl1, doi:10.1126/scisignal.2004088 (2013).

3 Cerami, E. *et al.* The cBio cancer genomics portal: an open platform for exploring multidimensional cancer genomics data. *Cancer discovery* **2**, 401-404, doi:10.1158/2159-8290.CD-12-0095 (2012).

Supplementary Figure S1

A.

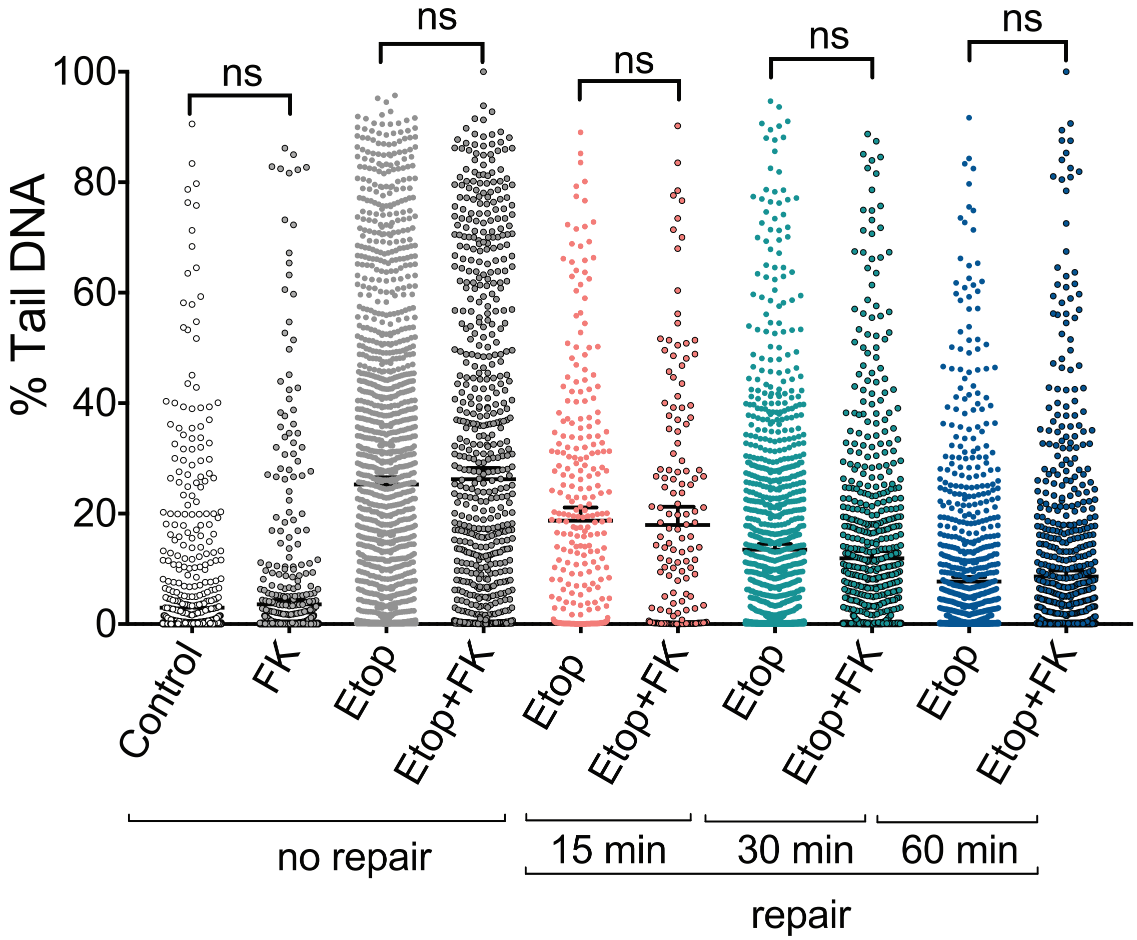

B.

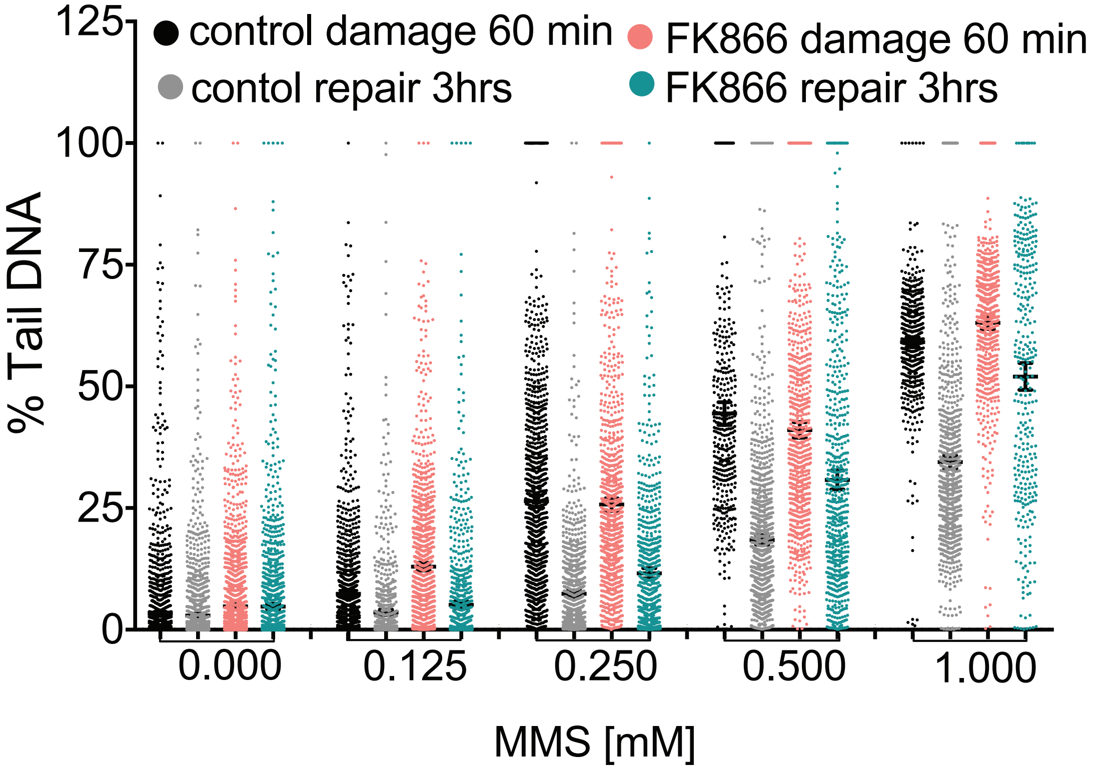

Supplementary Figure S2

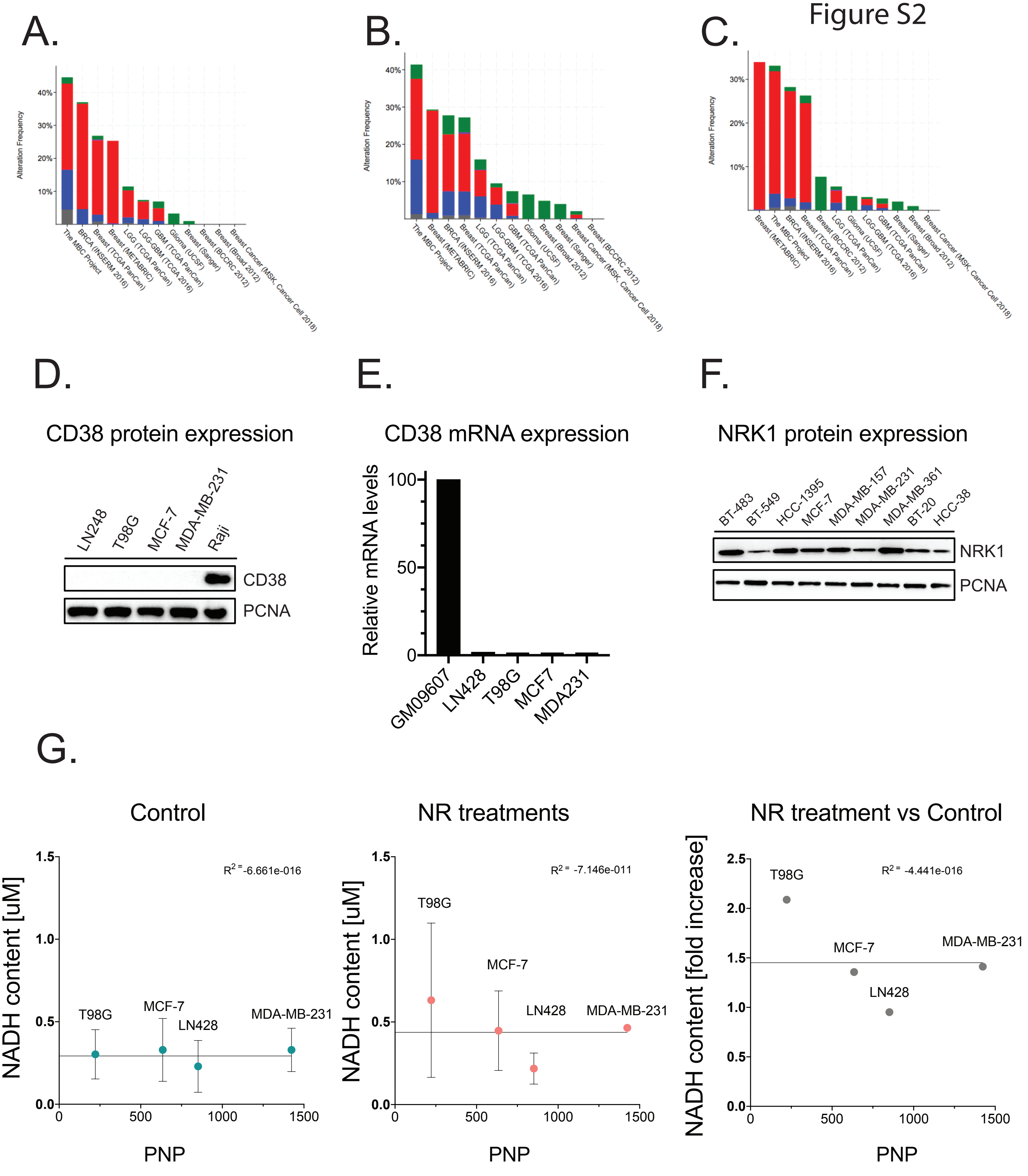

Supplementary Figure 3

A.

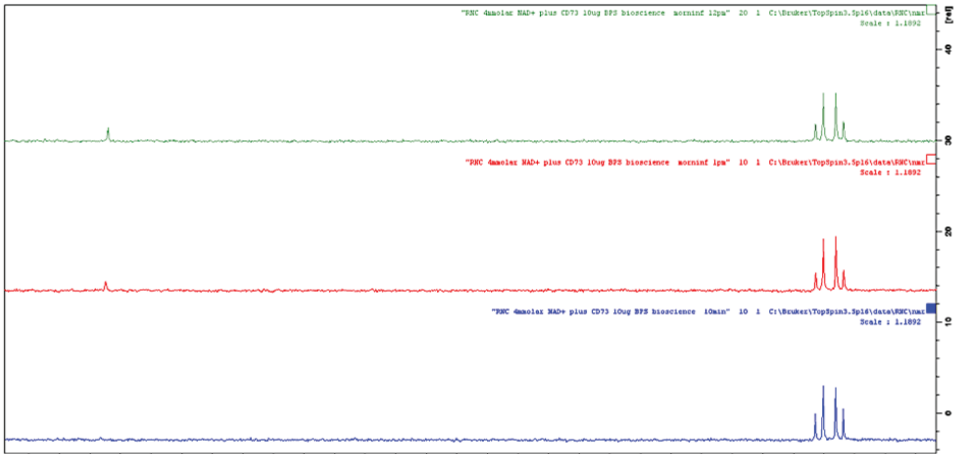

B.

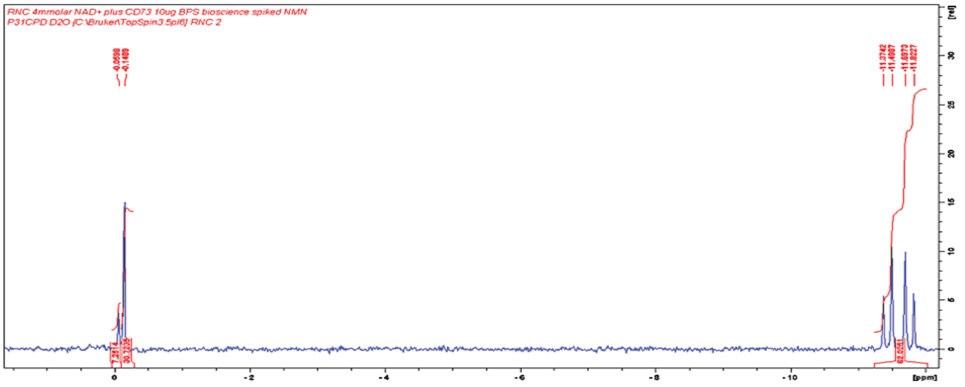

C.

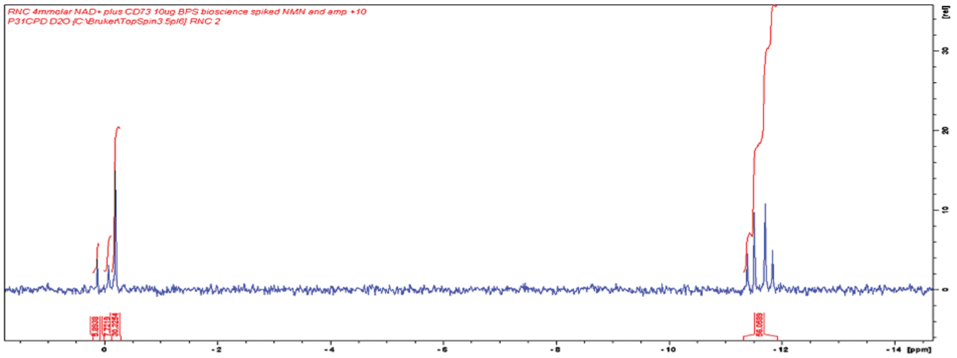

D.

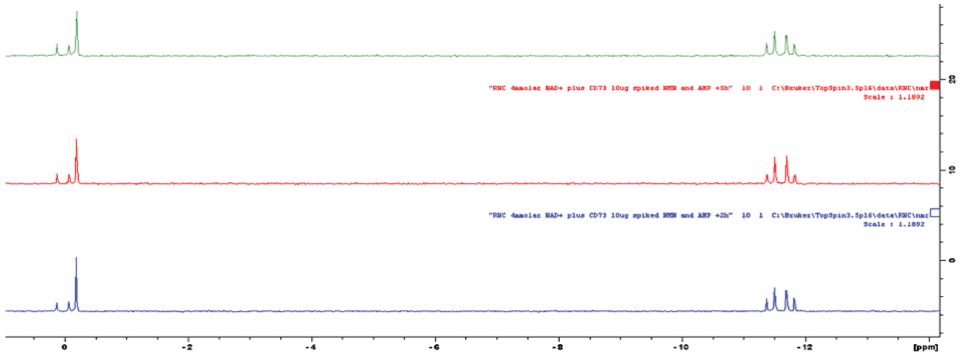

E.

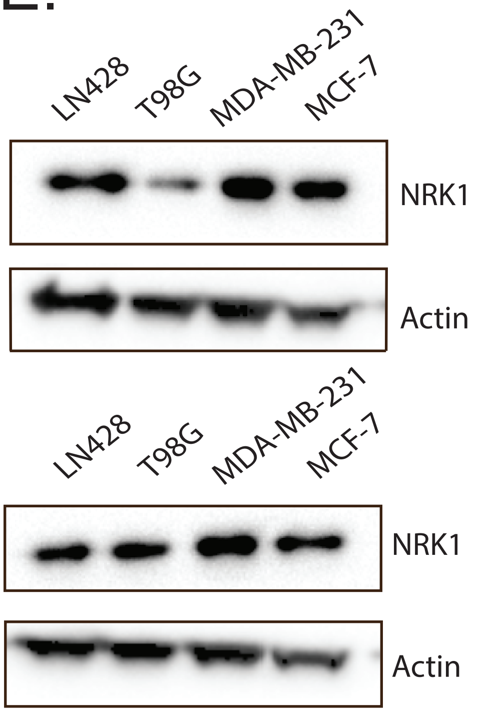

Supplemental Figure S4

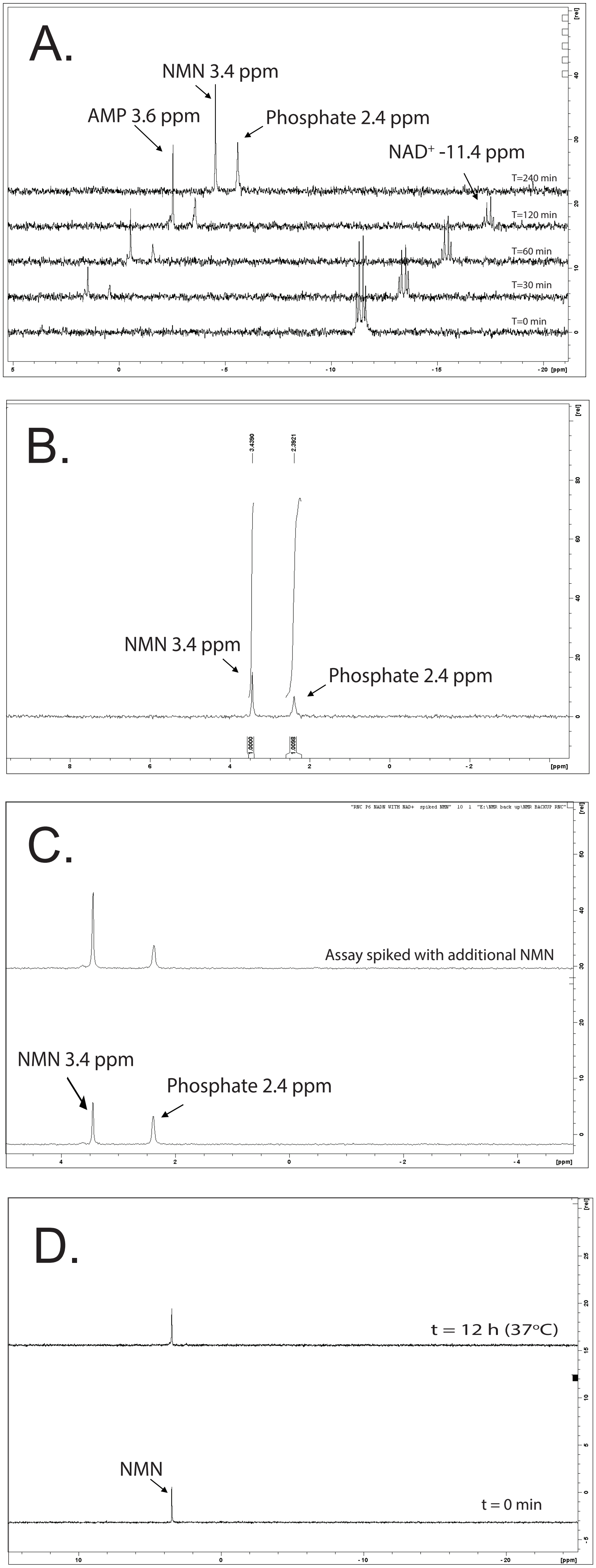

Supplementary Figure S5

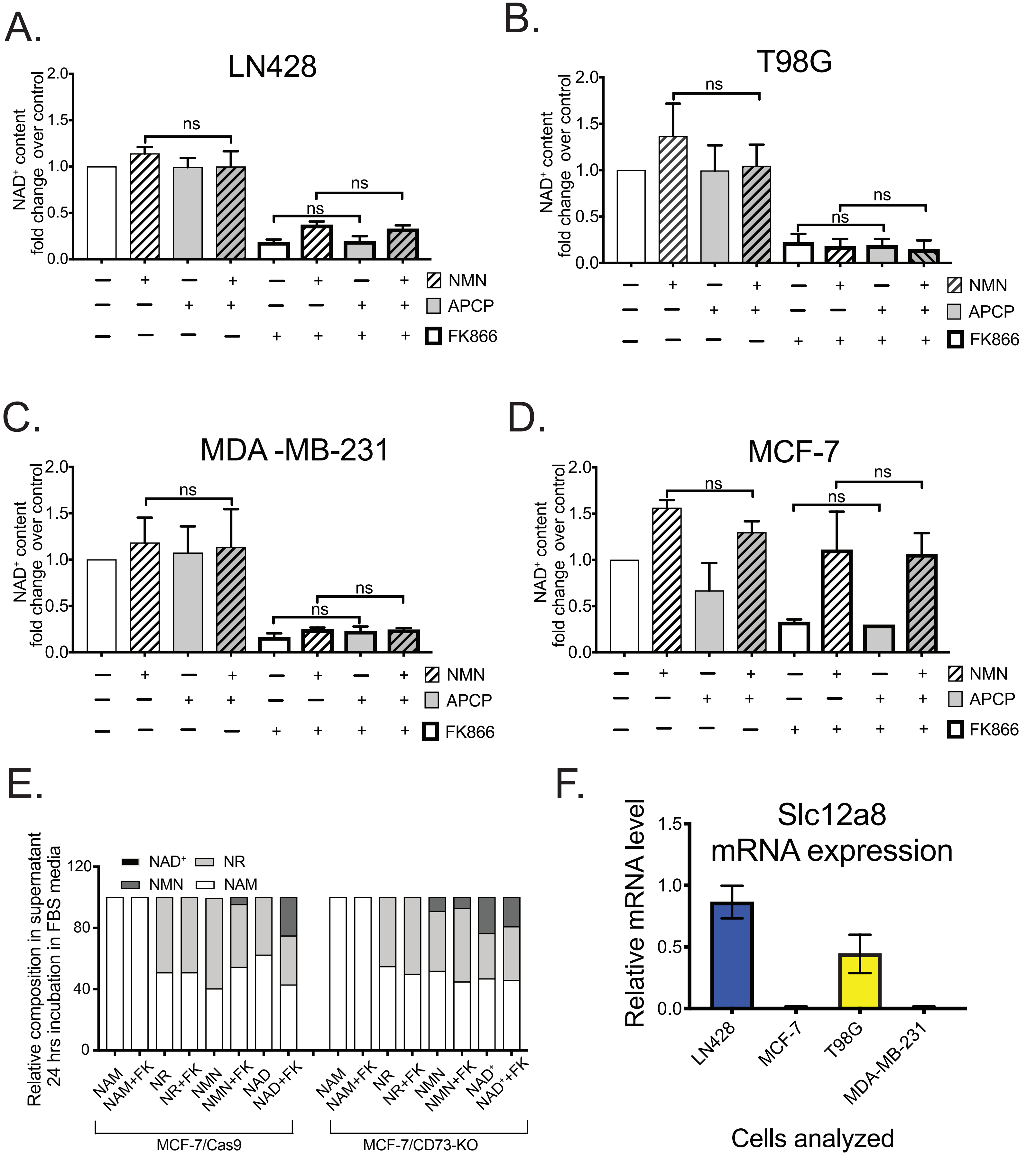

Supplementary Figure S6

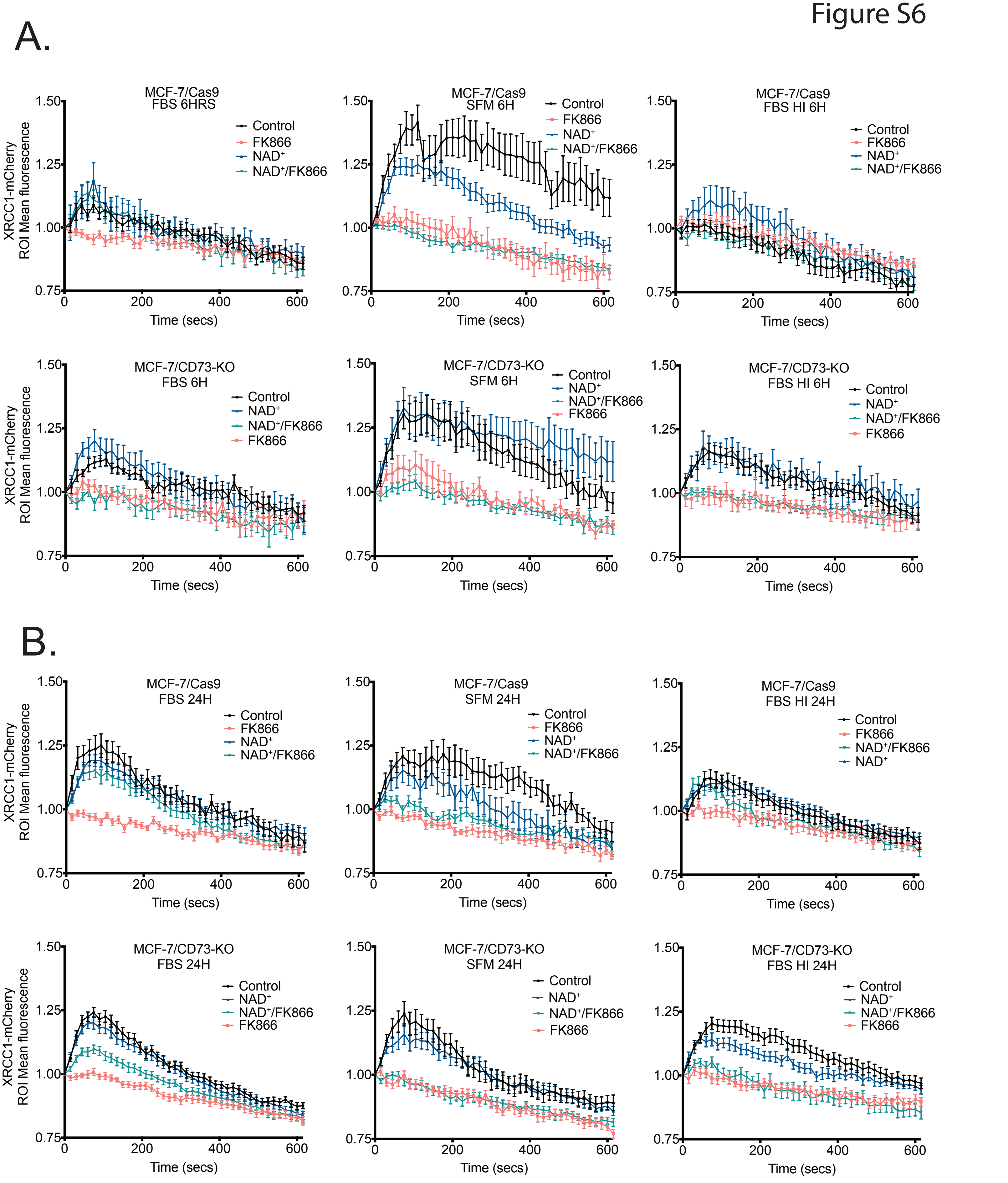

Supplementary Figure S7

A.

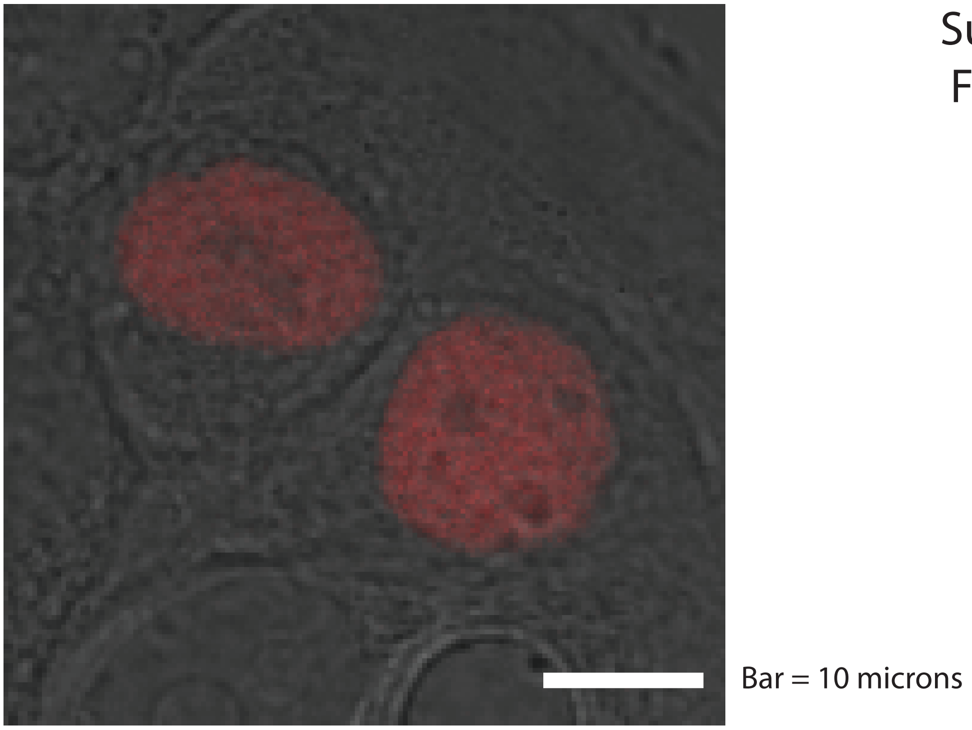

B.

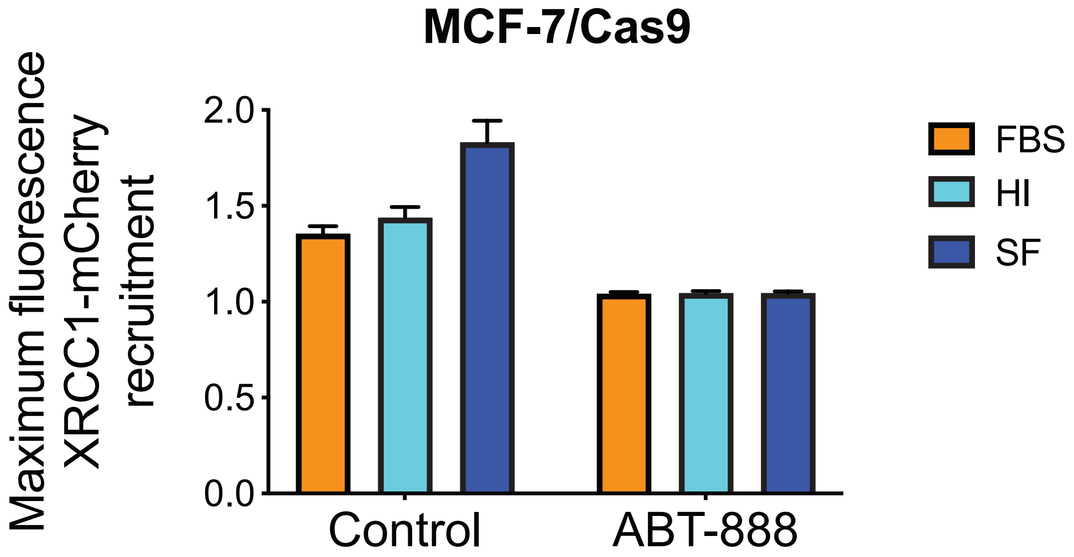

C.

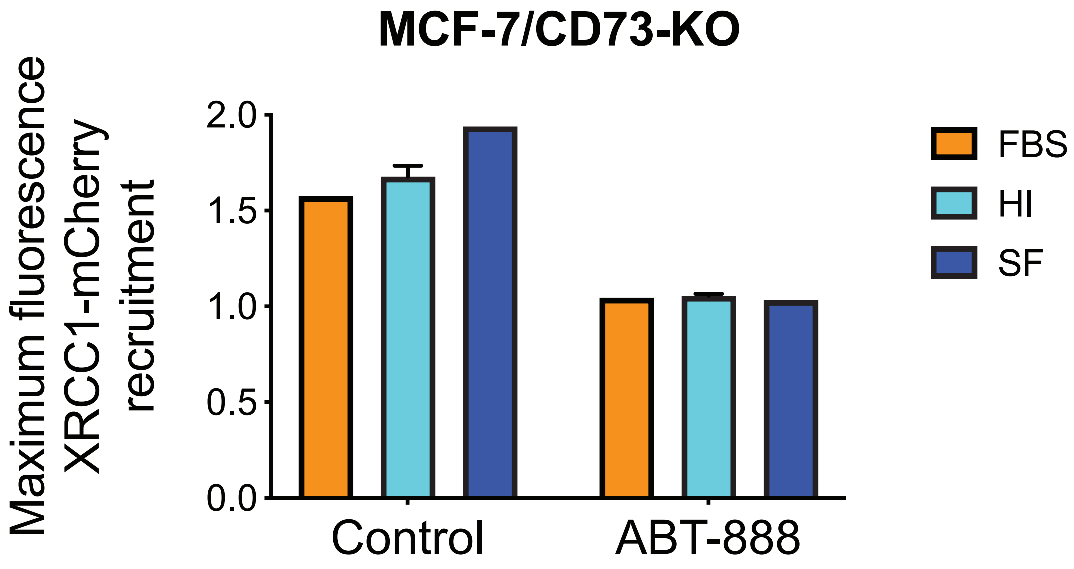

Supplementary Table S1. Human cell lines developed and/or used in this study

Cell line name	Cell line description	Growth media*
MDA-MB-231	Human breast cancer cell line	Media #1
BT-483	Human breast cancer cell line	Media #1
BT-549	Human breast cancer cell line	Media #1
HCC-1395	Human breast cancer cell line	Media #1
MCA-MB-157	Human breast cancer cell line	Media #1
MDA-MB-361	Human breast cancer cell line	Media #1
BT-20	Human breast cancer cell line	Media #1
HCC-38	Human breast cancer cell line	Media #1
T98G	Human glioblastoma cell line	Media #2
LN428	Human glioblastoma cell line	Media #3
293FT	Human embryonal kidney cells transformed with the SV40 large T antigen	Media #4
MCF-7	Human breast cancer cell line	Media #5
MCF-7/Cas9	MCF-7 cells expressing Cas9	Media #6
MCF-7/CD73-KO	MCF-7 cells with CD73 knockout by gRNA4	Media #7
MCF-7/Cas9/XRCC1-mcherry	MCF-7 cells expressing Cas9 and XRCC1mcherry	Media #8
MCF-7/CD73-KO/XRCC1-mcherry	MCF-7 cells with CD73 knockout by gRNA4 expressing XRCC1mcherry	Media #9
Raji	Human B lymphocyte cell line	Media #1

*Media #1: RPMI 1640 with 10% FBS (heat inactivated or not), 10 µg/ml Gentamycin.
*Media #2: EMEM with 10% FBS (heat inactivated or not), 1mM Sodium Pyruvate, 10mM MEM non-essential amino acids, Antibiotic/antimycotic, 5µg/mL Gentamycin.
*Media #3: Alpha EMEM with 10% FBS (heat inactivated or not), 2mM Glutamine, Antibiotic/antimycotic, 5µg/ml Gentamycin.
* Media #4: DMEM with 10% FBS (heat inactivated or not), 10mM MEM non-essential amino acids, Penicillin-Streptomycin, GlutaMax.
*Media #5: EMEM with 10% FBS (heat inactivated or not), 1mM Sodium Pyruvate, 10mM MEM non-essential amino acids, 10µg/ml human insulin solution.
*Media #6: Media #1 supplemented with Blasticidin (2.5µg/ml).
*Media #7: Media #1 supplemented with Puromycin(1.0µg/ml).
*Media #8: Media #1 supplemented with Blasticidin (2.0µg/ml) and Hygromycin (200µg/ml).
*Media #9: Media #1 supplemented with Puromycin(1.0µg/ml) and Hygromycin (200µg/ml).

Supplementary Table S2. Cell line authentication analysis

Sample	LabCorp SpecNbr	LabCorp CaseNbr	D3S1358	TH01	D21S11	D18S51	Penta E	D5S818	D13S317	D7S820	D16S539	CSF1PO	Penta D	vWA	D8S1179	TPOX	FGA	AMEL	Mouse
**LN428 (p9)**	85U90860	CX4005176	16, 17	8, 9.3	30, 31	13, 17	14, 16	11, 13, 14	8	8, 12	9	10	13	16	12, 13	8, 11	20, 25	X, Y	Not Det.
**MCF-7 (p8)**	85U90830	CX4005176	16	6	30	14	7, 12	11, 12	11	8, 9	11, 12	10	12	14, 15	10, 14	9, 12	23, 24, 25	X	Not Det.
**MDA-MB-231 (p10)**	85U90840	CX4005176	16, 17	7, 9.3	33.2	11	11	12	13	8, 9	12	12, 13	14	15, 18	13	8, 9	21, 22, 23	X	Not Det.
**T98G (p6)**	85U90850	CX4005176	16	7, 9.3	28, 32.2	13, 16	16	10, 12	13	9, 10	13	10, 12	10, 11	17, 20	13, 14	8	21, 22	X, Y	Not Det.

Supplementary Table S3. Vectors developed for and used in this study

Lab Stock #	Plasmid name	Insert description	Parental vector
263	pMDLg/pRRE	gag and pol; HIV lentiviral packaging plasmid	N/A
264	pMD2.g	VSV-G; HIV lentiviral packaging plasmid	N/A
266d	pRSVRev	Rev; HIV lentiviral packaging plasmid	N/A
727	pLVX-EGFP-IRES-Puro	EGFP	pLVX-IRES-GWB-Puro
1453	pLentiGuide-Puro	S. pyogenes sgRNA cassette	pLenti-Puro
1454	pLentiCas9-Blast	hSpCas9	pLenti-Blast
1494b	pLV-CMV-hXRCC1-mCherry-Hygro	Human XRCC1-mCherry fusion	pENTR/D-TOPO
1790	pLentiGuide-Puro/CD73(gRNA4)	CD73-specific gRNA, specific to exon 4	pLentiGuide-Puro
